# Supplementary material for: Woody species composition and diversity of agroforestry homegardens along altitudinal gradient in southwest Ethiopia
Source: PLoS One. 2025 Jan 7;20(1):e0313520. doi: 10.1371/journal.pone.0313520 (PMC11706438; doi:10.1371/journal.pone.0313520)
Supplement: S1 File — (DOC) [file pone.0313520.s001.doc]

Appendix

Appendix 1: List of woody species scientific, local and family name, and their origin in homegarden agroforestry systems of southwest Ethiopia

| **N^o^** | **Scientific name** | **Local name** | **Family name** | **Origin** |
| --- | --- | --- | --- | --- |
| 1 | *Acacia abyssinica* Hochst. ex Benth. | Sondii | Fabaceae | I |
| 2 | *Albizia gummifera* (J. F. Gmel.) C.A. Sm. | Ambabbessa | Fabaceae | I |
| 3 | *Albizia schimperiana* Oliv. | Ambabbessa arba | Fabaceae | I |
| 4 | *Allophylus abyssinicus* (Hochst.) Radikofer | Sehoo | Sapindaceae | I |
| 5 | *Annona senegalensis* pers. | Gishxaa | Annonaceae | I |
| 6 | *Artocarpus heterophyllus* | Jakifiruttii | Moraceae | E |
| 7 | *Azadirachta indica* A.Juss. | Niimii | Meliaceae | E |
| 8 | *Borassus aethiopum* Mart. | Meexxii | Arecaceae | I |
| 9 | *Brucea antidysenterica* J.F.Miller | Qomonyo | Simaroubaceae | I |
| 10 | *Calpurnia aurea* (Ait.) Benth. | Ceekaa | Fabaceae | E |
| 11 | *Carica papaya* L. | Papayee | Caricaceae | I |
| 12 | *Casimiroa edulis* LaLava | Kashimirii | Rutaceae | E |
| 13 | *Casuarina equisetifolia* | Shuwashuwe | Casuarinaceae | E |
| 14 | *Catha edulis* (Vahl) Forssk. ex Endl. | Caatii | Celastraceae | I |
| 15 | *Citrus aurantifolia* (Christm.) Swingle | Loomii | Rutaceae | E |
| 16 | *Citrus medica* | Marara | Rutaceae | E |
| 17 | *Citrus sinensis* | Burtukana | Rutaceae | E |
| 28 | *Coffea arabica* L | Buna | Rubiaceae | I |
| 19 | *Cordia africana* Lam. | Waddeessa | Boraginaceae | I |
| 20 | *Croton macrostachyus* Hochst ex Del. | Bakkannisa | Euphorbiaceae | I |
| 21 | *Cupressus lusitanica* | Gattira faranjii | Cupressaceae | E |
| 22 | *Dovyalis abyssinica* (A. Rich.) Warb. | Koshimii | Flacourtiaceae | I |
| 23 | *Dracaena fragrans* | Omo | Dracaenaceae | I |
| 24 | *Dracaena steudneri* Engl. | Bubbiftu | Dracaenaceae | I |
| 25 | *Duranta erecta* L. | Ababa | Verbenaceae | E |
| 26 | *Ehretia cymosa* Thonn. | Ulaaga | Boraginaceae | I |
| 27 | *Erythrina abyssinica* Lam. ex DC. | Waleensuu | Fabaceae | I |
| 28 | *Erythrina brucei* Schweinf. | Bero | Fabaceae | I® |
| 29 | *Eucalyptus camaldulensis* | Bargamo diimaa | Myrtaceae | E |
| 30 | *Eucalyptus citriodora* Hook. | Bargamo | Myrtaceae | E |
| 31 | *Eucalyptus globules* Labill. | Bargamo adii | Myrtaceae | E |
| 32 | *Ficus thonningii* Blume | Dambii | Moraceae | I |
| 33 | *Ficus sur* Forssk. | Harbuu | Moraceae | I |
| 34 | *Grevillea robusta* A.Cunn. ex R.Br. | Giravillaa | Proteaceae | E |
| 35 | *Jacaranda mimosifolia* D.Don | Jakaranda | Bignoniaceae | E |
| 36 | *Maesa lanceolata* Forssk. | Abbayyii | Myrsinaceae | I |
| 37 | *Malus domestica* | Appilii | Rosaceae | E |
| 38 | *Mangifera indica* L. | Mango | Anacardiaceae | E |
| 39 | *Millettia ferruginea* (Hochst.) Baker | Askira | Fabaceae | I® |
| 40 | *Moringa oleifera* | Moriinga | Moringaceae | E |
| 41 | *Morus alba* L. | Goraa | Moraceae | E |
| 42 | *Ocimum lamiifolium* Hochst. ex Benth | Damakase | Lamiaceae | I |
| 43 | *Olea europaea* (Wall. ex DC.) Cifferri | Ejersa | Oleaceae | I |
| 44 | *Olea welwitschii* (Knobl.) Gilg & Schellenb. | Baya | Oleaceae | I |
| 45 | *Persea americana* Mill. | Avokado | Lauraceae | E |
| 46 | *Pouteria campechiana* | Eggifiruttii | Sapotaceae | E |
| 47 | *Prunus persica* (L.) Batsch | Kukko | Rosaceae | E |
| 48 | *Psidium guajava* L. | Zetuna | Myrtaceae | E |
| 49 | *Rhamnus prinoides* L'Herit | Gesho | Rhamnaceae | I |
| 50 | *Ricinus communis* L. | Qobbo | Euphorbiaceae | I |
| 51 | *Sapium ellipticum* (Hochst) Pax. | Bosoqa | Euphorbiaceae | I |
| 52 | *Sesbania sesban* | Sasbaniya | Fabaceae | I |
| 53 | *Spathodea campanulata* | Ispatoda | Bignoniaceae | E |
| 54 | *Vernonia amygdalina* Del. | Eebicha | Asteraceae | I |
| 55 | *Vernonia auriculifera* Heirn. | Reejjii | Asteraceae | I |

**Key**: Origin: I- Indigenous, E- Exotic, ®- Endemic species

Appendix 2: Percentage frequency (%Freq), relative dominance (Rdo), relative density (Rde), relative frequency (RF) and importance value index (IVI) of woody species across the entire homegarden agroforestry systetms

| **N/S** | **Scientific Name** | **% Freq** | **Rdo** | **Rde** | **RF** | **IVI** |
| --- | --- | --- | --- | --- | --- | --- |
| 1 | *Catha edulis* | 54.2 | 7.4 | 54.5 | 7.1 | 69.1 |
| 2 | *Coffea arabica* | 84.7 | 10.6 | 29.3 | 11.1 | 51.0 |
| 3 | *Persea americana* | 79.2 | 27.5 | 3.0 | 10.4 | 40.8 |
| 4 | *Cordia africana* | 44.4 | 11.2 | 1.1 | 5.8 | 18.1 |
| 5 | *Mangifera indica* | 44.4 | 9.0 | 1.0 | 5.8 | 15.8 |
| 6 | *Grevillea robusta* | 36.1 | 6.3 | 1.0 | 4.7 | 12.0 |
| 7 | *Psidium guajava* | 37.5 | 3.8 | 0.6 | 4.9 | 9.3 |
| 8 | *Citrus sinensis* | 34.7 | 3.9 | 0.7 | 4.6 | 9.2 |
| 9 | *Ricinus communis* | 40.3 | 1.2 | 1.5 | 5.3 | 8.0 |
| 10 | *Carica papaya* | 20.8 | 3.7 | 0.5 | 2.7 | 6.9 |
| 11 | *Albizia gummifera* | 27.8 | 2.4 | 0.4 | 3.6 | 6.4 |
| 12 | *Vernonia auriculifera* | 30.6 | 0.0 | 1.0 | 4.0 | 5.0 |
| 13 | *Cupressus lusitanica* | 18.1 | 1.2 | 0.7 | 2.4 | 4.3 |
| 14 | *Annona senegalensis* | 18.1 | 1.3 | 0.5 | 2.4 | 4.1 |
| 15 | *Duranta erecta* | 23.6 | 0.0 | 0.7 | 3.1 | 3.8 |
| 16 | *Croton macrostachyus* | 13.9 | 0.8 | 0.3 | 1.8 | 2.9 |
| 17 | *Erythrina brucei* | 11.1 | 0.8 | 0.3 | 1.5 | 2.6 |
| 28 | *Vernonia amygdalina* | 12.5 | 0.7 | 0.1 | 1.6 | 2.4 |
| 19 | *Jacaranda mimosifolia* | 6.9 | 1.3 | 0.1 | 0.9 | 2.3 |
| 20 | *Casimiroa edulis* | 11.1 | 0.7 | 0.2 | 1.5 | 2.4 |
| 21 | *Sesbania sesban* | 12.5 | 0.1 | 0.4 | 1.6 | 2.2 |
| 22 | *Albizia schimperiana* | 6.9 | 0.8 | 0.1 | 0.9 | 1.8 |
| 23 | *Millettia ferruginea* | 4.2 | 1.1 | 0.1 | 0.6 | 1.7 |
| 24 | *Calpurnia aurea* | 9.7 | 0.1 | 0.4 | 1.3 | 1.7 |
| 25 | *Rhamnus prinoides* | 6.9 | 0.0 | 0.2 | 0.9 | 1.1 |
| 26 | *Ehretia cymosa* | 4.2 | 0.4 | 0.1 | 0.6 | 1.0 |
| 27 | *Acacia abyssinica* | 1.4 | 0.7 | 0.1 | 0.2 | 0.9 |
| 28 | *Malus domestica* | 5.6 | 0.0 | 0.3 | 0.7 | 1.0 |
| 29 | *Erythrina abyssinica* | 5.6 | 0.1 | 0.1 | 0.7 | 0.9 |
| 30 | *Citrus aurantifolia* | 5.6 | 0.1 | 0.1 | 0.7 | 0.9 |
| 31 | *Olea europaea* | 4.2 | 0.2 | 0.1 | 0.6 | 0.8 |
| 32 | *Casuarina equisetifolia* | 2.8 | 0.4 | 0.0 | 0.4 | 0.8 |
| 33 | *Eucalyptus citriodora* | 2.8 | 0.2 | 0.1 | 0.4 | 0.7 |
| 34 | *Eucalyptus globulus* | 2.8 | 0.2 | 0.1 | 0.4 | 0.6 |
| 35 | *Dovyalis abyssinica* | 2.8 | 0.1 | 0.1 | 0.4 | 0.5 |
| 36 | *Azadirachta indica* | 2.8 | 0.1 | 0.0 | 0.4 | 0.5 |
| 37 | *Olea welwitschii* | 2.8 | 0.1 | 0.0 | 0.4 | 0.5 |
| 38 | *Citrus medica* | 2.8 | 0.1 | 0.0 | 0.4 | 0.5 |
| 39 | *Dracaena steudneri* | 1.4 | 0.3 | 0.0 | 0.2 | 0.5 |
| 40 | *Artocarpus heterophyllus* | 6.9 | 0.2 | 0.0 | 0.2 | 0.5 |
| 41 | *Maesa lanceolata* | 2.8 | 0.1 | 0.0 | 0.4 | 0.5 |
| 42 | *Moringa oleifera* | 1.4 | 0.2 | 0.0 | 0.2 | 0.4 |
| 43 | *Morus alba* | 2.8 | 0.0 | 0.0 | 0.4 | 0.4 |
| 44 | *Borassus aethiopum* | 1.4 | 0.2 | 0.0 | 0.2 | 0.4 |
| 45 | *Allophylus abyssinicus* | 1.4 | 0.2 | 0.0 | 0.2 | 0.4 |
| 46 | *Pouteria campechiana* | 1.4 | 0.1 | 0.0 | 0.2 | 0.3 |
| 47 | *Ficus sur* | 1.4 | 0.1 | 0.0 | 0.2 | 0.3 |
| 48 | *Dracaena fragrans* | 1.4 | 0.1 | 0.1 | 0.2 | 0.3 |
| 49 | *Ficus thonningii* | 1.4 | 0.1 | 0.0 | 0.2 | 0.3 |
| 50 | *Eucalyptus camaldulensis* | 1.4 | 0.1 | 0.0 | 0.2 | 0.3 |
| 51 | *Prunus persica* | 1.4 | 0.1 | 0.0 | 0.2 | 0.3 |
| 52 | *Spathodea campanulata* | 1.4 | 0.1 | 0.0 | 0.2 | 0.3 |
| 53 | *Sapium ellipticum* | 1.4 | 0.1 | 0.0 | 0.2 | 0.3 |
| 54 | *Ocimum lamiifolium* | 1.4 | 0.0 | 0.0 | 0.2 | 0.2 |
| 55 | *Brucea antidysenterica* | 1.4 | 0.0 | 0.0 | 0.2 | 0.2 |
