# Supplementary material for: Woody species composition and diversity of agroforestry homegardens along altitudinal gradient in southwest Ethiopia
Source: PLoS One. 2025 Jan 7;20(1):e0313520. doi: 10.1371/journal.pone.0313520 (PMC11706438; doi:10.1371/journal.pone.0313520)
Supplement: Attachment — Submitted filename: PONE-D-24-12537_review round 2.pdf [file pone.0313520.s004.pdf]

PLOS ONE

Woody species composition and diversity of agroforestry homegardens along altitudinal gradient in southwest Ethiopia

--Manuscript Draft--

**Manuscript Number:**	PONE-D-24-12537R1
**Article Type:**	Research Article
**Full Title:**	Woody species composition and diversity of agroforestry homegardens along altitudinal gradient in southwest Ethiopia
**Short Title:**	Woody species diversity of agroforestry homegardens
**Corresponding Author:**	Tefera Jegora Kepura, MSc<br>Jimma University College of Agriculture and Veterinary Medicine<br>Jimma, Ethiopia ETHIOPIA
**Keywords:**	Altitudinal change, species richness, wealth status, woody diversity
**Abstract:**	Homegarden agroforestry systems that integrate trees with agricultural practices are usually valued for the conservation of farm biodiversity. Despite the system having a significant conservation role, litle is known on woody species composition and diversity following the elevation belt of southwest Ethiopia. A complete enumeration of 72 homegardens (24 each from altitudinal gradient) was purposively selected for woody species inventory. A total of 55 woody species belonging to 31 families and 45 genera were recorded. Of which, 56.4% of woody species are indigenous and two are endemic to Ethiopia. Family Fabaceae was the most represented family with eight species. The highest species richness (42) was observed at high altitudes followed by 39 species at middle and 31 species at low altitudes but no significant difference between them. Species richness significantly ($P < 0.001$) increased with increasing wealth status. The overall richness distribution was 46, 40, and 27 across rich, medium, and poor wealth classes respectively. Shannon diversity index differed significantly between poor and rich households. Pearson correlation result shows a strong positive and significant correlation between richness and wealth status. The mean woody species density was 89.06±9.25 and 1236.22±131.42 per garden and hectare, respectively. Stem density was significantly higher ($P < 0.001$) in wealthier farms. We found that, in southwestern Ethiopia wealthier agroforestry farms can support more woody species diversity in homegardens.
**Order of Authors:**	Tefera Jegora Kepura, MSc
	Kitessa Hundera Geleta, Professor (PhD)
	Zerihun Kebebew Fole, Associate Professor (PhD)
	Adugna Eneyew Bekele, Associate Professor (PhD)
**Response to Reviewers:**	All issues raised by the reviewer and editor were presented as described in detail in the attached documents.
**Additional Information:**	
**Question**	**Response**
**Financial Disclosure**<br><br>Enter a financial disclosure statement that describes the sources of funding for the work included in this submission. Review the submission guidelines for detailed requirements. View published research articles from *PLOS ONE* for specific examples.	The author(s) received no specific funding for this work.

Powered by Editorial Manager® and ProduXion Manager® from Aries Systems Corporation

This statement is required for submission and **will appear in the published article** if the submission is accepted. Please make sure it is accurate.<br><br>**Funded studies**<br>Enter a statement with the following details:<br>• Initials of the authors who received each award<br>• Grant numbers awarded to each author<br>• The full name of each funder<br>• URL of each funder website<br>• Did the sponsors or funders play any role in the study design, data collection and analysis, decision to publish, or preparation of the manuscript?<br><br>Did you receive funding for this work?	
**Competing Interests**<br><br>Use the instructions below to enter a competing interest statement for this submission. On behalf of all authors, disclose any competing interests that could be perceived to bias this work—acknowledging all financial support and any other relevant financial or non-financial competing interests.<br><br>This statement is required for submission and **will appear in the published article** if the submission is accepted. Please make sure it is accurate and that any funding sources listed in your Funding Information later in the submission form are also declared in your Financial Disclosure statement.<br><br>View published research articles from *PLOS ONE* for specific examples.	The authors have declared that no competing interests exist.

**NO authors have competing interests**<br><br>Enter: *The authors have declared that no competing interests exist.*<br><br>**Authors with competing interests**<br><br>Enter competing interest details beginning with this statement:<br><br>*I have read the journal's policy and the authors of this manuscript have the following competing interests: [insert competing interests here]*<br><br>* typeset	
**Ethics Statement**<br><br>Enter an ethics statement for this submission. This statement is required if the study involved:<br><br>• Human participants<br>• Human specimens or tissue<br>• Vertebrate animals or cephalopods<br>• Vertebrate embryos or tissues<br>• Field research<br><br>Write "N/A" if the submission does not require an ethics statement.<br><br>General guidance is provided below. Consult the submission guidelines for detailed instructions. **Make sure that all information entered here is included in the Methods section of the manuscript.**	N/A

Powered by Editorial Manager® and ProduXion Manager® from Aries Systems Corporation

**Format for specific study types**<br><br>**Human Subject Research (involving human participants and/or tissue)**<br>• Give the name of the institutional review board or ethics committee that approved the study<br>• Include the approval number and/or a statement indicating approval of this research<br>• Indicate the form of consent obtained (written/oral) or the reason that consent was not obtained (e.g. the data were analyzed anonymously)<br><br>**Animal Research (involving vertebrate animals, embryos or tissues)**<br>• Provide the name of the Institutional Animal Care and Use Committee (IACUC) or other relevant ethics board that reviewed the study protocol, and indicate whether they approved this research or granted a formal waiver of ethical approval<br>• Include an approval number if one was obtained<br>• If the study involved *non-human primates*, add *additional details* about animal welfare and steps taken to ameliorate suffering<br>• If anesthesia, euthanasia, or any kind of animal sacrifice is part of the study, include briefly which substances and/or methods were applied<br><br>**Field Research**<br><br>Include the following details if this study involves the collection of plant, animal, or other materials from a natural setting:<br>• Field permit number<br>• Name of the institution or relevant body that granted permission	
**Data Availability**<br><br>Authors are required to make all data underlying the findings described fully available, without restriction, and from the time of publication. PLOS allows rare exceptions to address legal and ethical concerns. See the PLOS Data Policy and FAQ for detailed information.	Yes - all data are fully available without restriction

A Data Availability Statement describing where the data can be found is required at submission. Your answers to this question constitute the Data Availability Statement and **will be published in the article**, if accepted.<br><br>**Important:** Stating 'data available on request from the author' is not sufficient. If your data are only available upon request, select 'No' for the first question and explain your exceptional situation in the text box.<br><br>Do the authors confirm that all data underlying the findings described in their manuscript are fully available without restriction?	
**Describe where the data may be found in full sentences. If you are copying our sample text, replace any instances of XXX with the appropriate details.**<br><br>• If the data are **held or will be held in a public repository**, include URLs, accession numbers or DOIs. If this information will only be available after acceptance, indicate this by ticking the box below. For example: *All XXX files are available from the XXX database (accession number(s) XXX, XXX.).*<br>• If the data are all contained **within the manuscript and/or Supporting Information files**, enter the following: *All relevant data are within the manuscript and its Supporting Information files.*<br>• If neither of these applies but you are able to provide **details of access elsewhere**, with or without limitations, please do so. For example:<br><br>*Data cannot be shared publicly because of [XXX]. Data are available from the XXX Institutional Data Access / Ethics Committee (contact via XXX) for researchers who meet the criteria for access to confidential data.*<br><br>*The data underlying the results presented in the study are available from (include the name of the third party*	All data are contained within the manuscript

and contact information or URL).<br>• This text is appropriate if the data are owned by a third party and authors do not have permission to share the data.<br><br>* typeset	
Additional data availability information:	Tick here if the URLs/accession numbers/DOIs will be available only after acceptance of the manuscript for publication so that we can ensure their inclusion before publication.; Tick here if your circumstances are not covered by the questions above and you need the journal's help to make your data available.

Manuscript Click here to access/download;Manuscript;Manuscript.doc

Woody species composition and diversity of agroforestry homegardens along altitudinal gradient in southwest Ethiopia

Tefera Jegora[1, 2,*], Kitessa Hundera[1], Zerihun Kebebew[1]and Adugna Eneyew[1]

[1]College of Agriculture and Veterinary Medicine, Jimma University, Ethiopia; [2]College of Agriculture and Forestry, Mettu University, Ethiopia, *- Corresponding author e-mail: teferajegora@gmail.com (TJ)

Abstract

Homegarden agroforestry systems that integrate trees with agricultural practices are usually valued for the conservation of farm biodiversity. Despite the system having a significant conservation role, litle is known on woody species composition and diversity following the elevation belt of southwest Ethiopia. A complete enumeration of 72 homegardens (24 each from altitudinal gradient) was purposively selected for woody species inventory. A total of 55 woody species belonging to 31 families and 45 genera were recorded. Of which, 56.4% of woody species are indigenous and two are endemic to Ethiopia. Family Fabaceae was the most represented family with eight species. The highest species richness (42) was observed at high altitudes followed by 39 species at middle and 31 species at low altitudes but no significant difference between them. Species richness significantly ($P < 0.001$) increased with increasing wealth status. The overall richness distribution was 46, 40, and 27 across rich, medium, and poor wealth classes respectively. Shannon diversity index differed significantly between poor and rich households. Pearson correlation result shows a strong positive and significant correlation between richness and wealth status. The mean woody species density was 89.06±9.25 and

1236.22±131.42 per garden and hectare, respectively. Stem density was significantly higher ($P < 0.001$) in wealthier farms. We found that, in southwestern Ethiopia wealthier agroforestry farms can support more woody species diversity in homegardens.

Key words: Altitudinal change, species richness, wealth status, woody diversity

Background

Agroforestry is a strategic land use plan to conserve biodiversity in deforested and fragmented landscapes [1-3]. The practice integrates the most environmentally sound forestry and agricultural systems into the same land management unit with sustainable and profitable interaction between trees, crops, and livestock [4]. It supports the floral and faunal assemblages that can be as species-rich, abundant, and diverse as forests with modified and non-forest species compositions [5, 6]. These help agroforestry systems to have diverse components and multi-strata structures with multiple ecological and socio-economic benefits [7-9].

Agroforestry is a potential biodiversity conservation site following intact natural forests as it provides an important reservoir for terrestrial biodiversity [10, 11]. Tropical agroforestry systems have high levels of woody species diversity that are naturally retained and/or planted on farmland [12-14]. A diversified woody species in the homegarden agroforestry systems of southwest Ethiopia is one of these dominant practices, which is dominated by khat and coffee shrubs. *Coffea arabica* and *Catha edulis* are the two most important income-generting woody species of homegardes in southern Ethiopia [15]). It encourages tree plantation and maintenance to diversify multipurpose woody species that support smallholder farmers' livelihoods [16-18]. Agroforestry systems serve as in situ conservations containing high species richness and

diversity valued and conserved by the farmers [10, 19]. As a result, agroforestry plays five vital roles in biodiversity conservation: (1) provides habitat for species to tolerate a certain level of disturbance; (2) reduces the rate of conversion of natural habitat by providing more productive and sustainable alternative agricultural systems; (3) provides corridors between habitat remnants and the conservation of area-sensitive floral and faunal species; (4) preserves the germplasm of sensitive species; and (5) provides ecosystem services such as erosion control, increased infiltration capacity, reduced degradation, and habitat fragmentation [3, 20-22].

Homegarden agroforestry is usually managed by members of the households for the production of subsistence crops and sometimes cash objectives [23]. The diverse and multi-strata components of the system not only provide significant food and income sources [24-27] but also serve as biodiversity conservation [28, 29]. It varies from the nearby agroforestry practices due to their mosaic patches or plots dominated by a diversity of woody species [15]. Consisting of multiple strata, complex structure, high species richness, evenness, and abundance make homegardens typically similar to natural forests [6, 30]. Homegarden contains high species diversity and usually 3–4 vertical canopy strata, including trees as an upper story, herbaceous vegetation as near ground, and intermediate layers in between [4, 7, 15]. This composite nature of the agroforestry system is under the influence of different ecological and socio-economic factors globally [14, 31, 32]. For example, [33] revealed that household level of resource endowment significantly affects tree number, species richness, and species diversity.

In Ethiopia, agroforestry is an old farming system containing low to high woody species diversity distribution due to variations in ecological and socio-economic factors such as

elevation, size of farmland, and wealth status [15, 25, 34-36]. The results of the present study did not show a significant difference among altitudinal gradients and woody species distribution. However, household wealth variation significantly influences the distribution of woody species composition and diversity in homegarden agroforestry systems. Woody species composition in the agroforestry system is influenced by household wealth status and other socio-economic attributes such as size of farmland, level of education, family size, and off-farm economic sources [37]. In indigenous agricultural practices of the northern Ethiopian homegardens, [38] reported variations between woody species composition, density, and diversity along altitudinal gradients. Additionally, [39] recommends further investigation into the interaction between homegarden agroforestry woody species and their physical environment in southwestern Ethiopia. Therefore, this study was conducted to reveal woody species composition and diversity of homegarden agroforestry systems along an altitudinal gradient and household wealth status in southwest Ethiopia.

Materials and methods

Description of the study area

The study was conducted in three districts, namely Mana, Gomma, and Dhidhessa, following the elevation belt of southwest Ethiopia. The area is located between 7°40ˈN-8°10ˈN and 36°30ˈE-36°50ˈE with an elevation range of 1459m to 2542m above sea level (Fig 1). The area received a mean annual rainfall of 1570mm with minimum and maximum temperature of 13–28 °C, respectively. It is occupied by cultivated land, wetlands, settlements, commercial farms, shrubland, grassland, and forest land. Agro-ecologically, it is classified into lowland, midland,

and highland, with midland-dominated areas. Agriculture is the main source of socio-economic development, with coffee and khat as dominant cash crops. Homegarden agroforestry (coffee, enset and khat)-based systems are the most accredited farming systems.

Fig 1. Map of the study area. The map was created using shapefiles acquired for free from the Ethiopian Mapping Agency. The Digital Elevation Model (SRTM, 30m resolution) can be accessed for free at EarthExplorer (usgs.gov)

Sampling strategy

Woody species inventory was conducted in homegarden agroforestry systems across the elevation belt of southwest Ethiopia. Elevation classification was made purposively based on the presence of homegarden agroforestry systems. Elevation data was gathered from the Digital Elevation Model in Google Earth [40]. Then, three altitudinal gradients were made as low (1459–1750 m), middle (1750–2050 m), and high (2050–2542 m) following the distributions of homegarden agroforestry with its perspective altitude to see the effect of altitudinal change on woody species diversity. Additionally, wealth status was used as it positively or negatively affects woody species in homegarden agroforestry systems and is classified as rich, medium and poor [14, 41]. The classification was made with the assistance of the developmental agents and local elders. Here, the developmental agent refers to the agricultural extension worker at the smallest administrative unit (locally called kebele) in Ethiopia. Local criteria such as the number of livestock holdings, total size of land holdings, housing standard and annual consumption of food and sell crop production were used for this classification. Then, a complete homegarden

enumeration was made for the woody species inventory [42]. Finally, a total of 72 homegardens (24 from each altitudinal gradient) were purposively selected for woody species inventory.

Methods of data collection

Woody species with a diameter at breast height (DBH) ≥5cm and height ≥1.5m were identified and their height and DBH were measured. Individuals below this measurement were only counted and registered. For woody branched below 1.3m above the ground, the average DBH was calculated from all branches measured separately [36]. Tree DBH and height were measured using a caliper and a Sunto Clinometer respectively. Tree size beyond the caliper measurement was measured using mater tape and converted to diameter. Tree height difficult to measure with a Sunto Clinometer was measured by a marked range pole. The altitudinal gradient was measured using the Global Positioning System (GPS) Garmin 72. Woody species identification was made on the field and voucher specimen. A voucher specimen was collected, pressed, dried and identified following [43] and flora of Ethiopia [44-46].

Data analysis

Woody species diversity was analyzed using diversity indices [47-49]. Shannon Wiener diversity index which takes species richness and evenness into consideration was calculated as:

$$H' = -\sum_{i=1}^{s} pilnpi \ldots\ldots\ldots (1)$$

Where: H' = Shannon-Wiener diversity index, s = number of species and Pi = the proportion of individuals of the ith species and ln =s natural logarithm.

A measure of similarity of the abundances of different woody species in the sampled area was analyzed using Evenness/Equitability index.

$$E = \frac{H'}{Hmax} = \frac{H'}{lnS} \ with\ Hmax = lnS \ \ldots\ldots\ldots \ (2)$$

Where: E = Evenness (Equitability), Hmax = ln(S) is the maximum level of diversity possible within a given population.

The similarity in species composition of the two sites is computed using Sorensen's Similarity Coefficient (S_s) with values ranging between 0 and 1: 0 indicates complete dissimilarity and 1 complete similarity.

$$Ss = \frac{2a}{2a+b+c} \ldots\ldots\ldots \ (3)$$

Where: Ss = Similarity index, a = number of species common in the two sites; b = number of species recorded only in site one; and c = number of species recorded only in site two.

Importance value index (IVI) is a useful ecological parameter to compare the environmental significance of woody species. IVI was calculated using the sum of relative density, frequency and dominance as:

IVI= Relative density + Relative dominance + Relative frequency ……………………… (4)

Where: $\text{Relative Density (RDe)} = \frac{\text{Stem count of a species}}{\text{Stem count of all species}} * 100$ ………………………..…4.1

$$\text{Relative Dominance (RDo)} = \frac{\text{Doninance of a species}}{\text{Dominance of all species}} * 100 \quad \ldots\ldots\ldots\ldots\ldots\ldots\ldots 4.2$$

$$\text{Relative Frequency (RF)} = \frac{\text{Frequency of a species}}{\text{Frequency of all species}} * 100 \quad \ldots\ldots\ldots\ldots\ldots\ldots\ldots 4.3$$

The density of woody species was calculated by converting the total number of individuals encountered in all plots to equivalent number per hectare using the following formula:

$$D = N/S \quad \ldots\ldots\ldots\ldots\ldots\ldots (5)$$

Where: D - the density (stems ha^{-1}), N - the number of stems present on the considered surface and S - the area considered in hectare (ha).

Basal area, the area occuied by woody species was calculated for all woody species DBH ≥5cm using the following formula:

$$\text{Basal area (BA)} = \pi d^2/4 \quad \ldots\ldots\ldots\ldots\ldots\ldots (6)$$

Where: π= 3.14; d = diameter at breast height (m)

Statistical analysis was made using R software version 4.1.3 [50]. Homogeneity and normality of the variance were approved using the LeveneTest and Shapiro-Wilk tests in R statistics. Non-normally distributed data was analyzed using the log transform and the removal of the outlier. The variables were compared using one-way ANOVA (analysis of variance). The relationship between altitudinal gradients and wealth status and the diversity indices was seen using Pearson's correlation coefficient test. The least significant difference (LSD) test was used to compare the level of significance ($P < 0.05$) between each variable.

Results

Floristic composition

In total, 55 woody species belonging to 31 families and 45 genera were recorded in homegarden agroforestry systems. Family Fabaceae is the most dominant having eight species followed by Moraceae, Myrtaceae and Rutaceae each with four species. About 34.5% of the remaining families had only one woody species. About 56.4% of species are indigenous including two endemics (*Erythrina brucei* and *Millettia ferruginea*), and the remaining 43.6% are exotic (Appendix 1). The species richness was 31, 39 and 42 for low, middle and high altitudes, respectively. Woody species richness increases with increasing wealth status. Accordingly, 27, 40 and 46 species were recorded for poor, medium and rich households respectively (Table 1). Species richness was not significant ($P < 0.05$) between altitudinal gradients, whereas it significantly ($P < 0.001$) increased with increasing wealth status (Table 2). The percentage frequency occurrence of woody species lay between 1.4 - 84.7%. *Coffea arabica* (84.7%), *Persea americana* (79.5%), and *Catha edulis* (54.2%) are the most frequently observed species. However, about 15 species were observed only once in surveyed homegardens (Appendix 2).

Table 1: Overall woody species richness, genera and family distiribution of woody species across altitudinal gradient and wealth status in homegarden agroforestry systems

**Category**	**Number**	**Richness**	**Genus**	**Family**
**Altitudinal gradient**				

**Low**	24	31	26	21
**Middle**	24	39	36	25
**High**	24	42	37	25
**Total**	72	55	45	31
**Wealth class**				
**Poor**	24	27	24	21
**Medium**	24	40	33	24
**Rich**	24	46	39	27
**Total**	72	55	45	31

Table 2: Species richness, Shannon diversity index and Evenness (Mean ± SE) of woody species across altitudinal gradients and wealth status in southwest Ethiopia

Category	Richness	Shannon	Evenness
**Altitudinal gradients**			
**Low**	$7.54^{a}\pm0.59$	$1.00^{a}\pm0.06$	$0.53^{a}\pm0.03$
**Middle**	$7.42^{a}\pm0.77$	$1.07^{a}\pm0.09$	$0.57^{a}\pm0.04$
**High**	$7.75^{a}\pm0.76$	$0.97^{a}\pm0.08$	$0.55^{a}\pm0.05$

**Mean**	7.57±0.40	1.02±0.05	0.55±0.02
**Wealth status**			
**Poor**	4.75^{c}±0.42	0.84^{b}±0.06	0.61^{a}±0.04
**Medium**	7.58^{b}±0.46	1.07^{a}±0.09	0.53^{a}±0.04
**Rich**	10.38^{a}±0.66	1.14^{a}±0.07	0.50^{a}±0.03
**Mean**	7.57±0.40	1.02±0.05	0.55±0.02

Key- Different letters superscripted along column mean value indicate significant different ($P < 0.05$), SE- standard error

Woody species diversity

The Shannon diversity index and evenness/equitability index were analyzed along an altitudinal gradient and wealth status. The Shannon and evenness were not significantly different ($P < 0.05$) between altitudinal gradients. The highest mean Shannon (1.07±0.09) and evenness (0.57±0.04) indexes were observed at middle altitude. The lowest Shannon (0.97±0.08) was recorded at high altitudes, whereas the lowest evenness (0.53±0.03) was recorded at low altitudes. Species richness and mean Shannon diversity index increased with household wealth status, whereas evenness showed the reverse. The Shannon diversity index was significant ($P < 0.05$) between poor and rich households, while evenness didn't show a significant difference between wealth (Table 2). The Pearson correlation result showed no relation between species richness and altitude, and a positively significant correlation was seen between species richness and wealth

status. Furthermore, the Shannon diversity index was significantly and positively correlated to wealth status (Table 3).

Table 3: Pearson correlation between altitudinal gradient, wealth status and woody species diversity in homegarden agroforestry systems

**Category**	**Altitudinal gradients**	**Wealth status**	**Species richness**	**Shannon diversity index**
**Wealth status**	0.000	-	-	-
**Species richness**	0.025	0.674^{**}	-	-
**Shannon diversity index**	-0.028	0.319^{**}	0.470^{**}	-
**Evenness**	0.055	-0.225	-0.270^{*}	0.610^{**}

Note: *- significant different ($P < 0.05$), **- significant different ($P < 0.01$)

Woody species similarity index

The calculated similarity index result indicates that about 51% of the woody species are similar along altitudinal gradients and wealth classes (Fig 2). The highest similarity index was observed between high and middle altitudes (55%) and the lowest (45%) was registered between high and low altitudes. For a wealth class, the highest (60%) similarity was seen between high and medium classes while the lowest (40%) was seen between medium and poor classes (Table 4). On the other hand, 12, seven and three woody species were unique to high, middle and low

altitudes respectively, whereas 10, six and two species were recorded for rich, medium and poor households respectively (Fig 2).

Fig 2. Venn diagram representation of the similarity and dissimilarity of woody species across (a) wealth status and (b) altitudinal gradient in homegarden agroforestry systems

Table 4: Woody species similarity index in homegarden agroforestry systems across altitudinal gradient and wealth status

**Altitudinal gradient**	**High**	**Middle**	**Low**
**Middle**	0.55	-	-
**Low**	0.45	0.47	-
**Wealth status**	**Rich**	**Medium**	**Poor**
**Medium**	0.60	-	-
**Poor**	0.44	0.40	-

Woody species density

Woody species stem density was calculated along an altitudinal gradient and wealth status. The mean stem density per homegarden and hectare was 89.06±9.25 and 1236.22±131.42 respectively. The mean stem density was significantly different ($P < 0.001$) between wealth classes. The highest 134.88±18.87 stem density was recorded in the rich followed by medium

87.08±14.35 and poor 45.21±7.38 wealth classes. However, there is no significant difference between stem density and altitudinal gradient at the garden and hectare level (Table 5).

Table 5: Mean (±SE) woody density and basal area distribtution along altitudinal gradients and wealth status in homegarden agroforestry systems

Category	Density/garden	Density/ha	BA/garden	BA/ha
**Altitudinal gradients**				
**Low**	82.04^{a}±14.44	1151.88 ^{a}±140.63	0.64^{a}±0.13	8.97^{a}±1.49
**Middle**	79.88^{a}±12.10	1298.50^{a}±275.63	1.28^{a}±0.31	15.00^{a}±3.49
**High**	105.25^{a}±20.48	1258.29^{a} ±252.41	0.84^{a}±0.28	11.29^{a}±3.79
**Wealth status**				
**Poor**	45.21^{c}±7.38	1654.29^{a}±268.98	0.33^{b}±0.08	10.59 ^{a}±2.71
**Medium**	87.08^{b}±14.35	1046.13^{ab}±169.67	0.90^{ab}±0.27	11.37 ^{a}±1.70
**Rich**	134.88^{a}±18.87	1008.25^{b}±217.68	1.53^{a}±0.29	12.09 ^{a}±3.65

Key: Different letters superscripted along column mean value indicate significant different ($P < 0.05$), SE- standard error

Basal area distribution

The mean basal area was highest at middle altitude followed by high and low altitude respectively, although there was no significant difference between each altitude. Homegarden level basal area was 1.53±0.29 for the rich, 0.90±0.27 for the medium, and 0.33±0.08 for the poor in which a significant difference ($P < 0.01$) was seen between the poor and rich classes (Table 5). However, there was no significant difference in basal area per hectare (m^2 ha^{-1}) along elevation gradients and wealth status. Finally, the overall basal area distributions of the top ten woody species are presented in Figure 3. *Persea americana* had the highest basal area (886.87 m^2 ha^{-1}) followed by *Cordia africana* (146.48 m^2 ha^{-1}), *Coffea arabica* (130.87 m^2 ha^{-1}), *Mangifera indica* (95.68 m^2 ha^{-1}), *Catha edulis* (64.98 m^2 ha^{-1}), *Grevillea robusta* (46.30 m^2 ha^{-1}), *Citrus sinensis* (18.01 m^2 ha^{-1}), *Psidium guajava* (16.79 m^2 ha^{-1}), *Carica papaya* (15.69 m^2 ha^{-1}) and *Albizia gummifera* (6.51 m^2 ha^{-1}) (Fig 3).

Fig 3. Basal area distribution (m^2 ha^{-1}) for the top ten woody species in homegarden agroforestry systems at southwest Ethiopia

Importance value index

The overall importance value index in the entire homegarden agroforestry system value lay between 0.20 to 69.06% for less to more important woody species respectively. However, a large number (29) of woody species had an importance value index of less than 1% (Appendix, 1). Figure 4 indicates the importance value index distribution of the top ten woody species. Accordingly, the importance value of these species was presented as *Catha edulis* (69.06%), *Coffea arabica* (51.01%), *Persea americana* (40.84%), *Cordia africana* (18.07%), *Mangifera*

indica (15.81%), *Grevillea robusta (11.98%), Psidium guajava (9.31%), Citrus sinensis (9.17%), Ricinus communis (7.97%)* and *Carica papaya* (6.87%) in their descending order (Fig 4).

Fig 4. Importance value index of the top ten woody species across the entire homegarden agroforestry systems

Discussion

Woody species composition and diversity significantly vary between wealth statuses but not between altitudinal gradients. Species richness and the Shannon diversity index have shown a significant increasing trend with increasing wealth status. The result was agreed with [14, 51], who reported increased richness and Shannon diversity index with increasing wealth status in south-central Ethiopia. Evenness had shown a reverse trend with richness in wealth classes. The result indicates that homegardens with few numbers of different species (lower species richness) have a large abundance for each species (higher evenness). However, there was no continuous increasing or decreasing values of diversity indices seen along altitudinal gradients. Likewise, species richness and diversity in agroforestry didn't show a parallel relationship with the elevation gradient [52]. *Catha edulis, Coffea arabica* and *Persea americana* are the most abundant and frequently recorded woody species. The study conducted by [38] reported that the distributions of farm woody species are determined by the economic or ecological values they provide. Therefore, the main reason for the higher abundance and distribution of these woody species may be due to the farmer's focus on the production of highly economic and ecologically valued woody species in the present study area.

The number of woody species recorded in the present study area is comparable to the number of woody species (55) reported in the traditional agroforestry practices of Dellomenna district, Ethiopia [53]. The number is lower than 120 trees and shrubs species reported from southern Ethiopia [15] and 60 woody species around Jimma Town, southwestern Ethiopia [39] within similar agroecology. It's also lower than 83 plant species in eastern Amazonia [54], 138 in south-central Ethiopia [14], 171 in central Nepal [55], and 206 in southern and southwestern Ethiopia [29]. However, it's higher than 53, 36, 49 and 32 woody species reported from Shewarobit district [56], Shashemene district [57], southern Ethiopia [58], and southern Tigray [28] in Ethiopia, respectively. It is also higher than 32 woody species in western Cameroon [59] and 30 woody species in the mid-highlands of southern Ethiopia [52].

The present study registered 56.4% indigenous and 43.6% exotic whereas 85% indigenous and 15% exotic were reported in Dellomenna district, southeastern Ethiopia [53]. This inverse result may be observed due to two main reasons. 1) Ecological difference between the two sites as Dellomenna is located in the forest-savannah transition zone of southern Ethiopia and the present study area located in Eastern Afromontane Forests dominated by woody plantation following the expansion of farmland in southwest Ethiopia. 2) Variation in farmers' preference ranking towards the selection of an exotic and native woody species. The present finding results agreed with the study conducted by [29] who reported 69.52% indigenous and 31.48% exotic in homegarden agroforestry systems from similar agroecology. Similar to this study, *Erythrina brucei* and *Millettia ferruginea* were also reported as the two endemic woody species in Ethiopia in traditional agroforestry practices [60].

The variation in the woody species similarity index recorded was not only because of elevation change but also wealth status which was affected by the farmer's homegarden size, extension service, improved seedlings, and labor forces. Likewise, the species similarity index between the sites changed with elevation and woody production [61]. The highest similarity index reported between high and middle altitudes agreed with [52], who reported the highest species similarity between adjacent elevations in coffee-based agroforestry in Ethiopia. Also, [59] reported the highest woody species similarity due to closed ecological conditions between coffee agroforestry systems and forests in Cameroon.

The mean stem density was significantly increased with wealth status but there was no significant difference between altitudinal gradients. This indicates that household wealth status determines woody species density in homegarden agroforestry systems. The present study reported the top ten woody species having the highest basal area. Of which, *Persea americana*, an exotic fruit tree species has the highest (61.52%) basal area. However, [51] reported that *Eucalyptus camaldulensis* had the highest (30.03%) basal area among the top ten woody species basal area registered in traditional agroforestry practices of Wondo district, south-central Ethiopia. The distribution indicates that *Cordia africana*, a native endangered tree occupies the second largest basal area which has been recorded on the fourth step among woody species having the highest importance value index. The highest importance value index was recorded for *Catha edulis*. Similarly, *Catha edulis* was recorded as an economically important woody crop in homegardens (Gebrehiwot *et al*., 2016). On the contrary, *Eucalyptus camaldulensis* [15, 51], *Faidherbia albida* [34], and *Cordia africana* [40] were reported as the most important woody species in homegarden agroforestry systems in Ethiopia.

Conclusion

Homegarden agroforestry systems in southwestern Ethiopia are rich in woody species diversity. Households within a rich wealth class had the highest number of woody species compared to medium and poor wealth classes. Woody species richness was not statistically significant between altitudinal gradients. Shannon diversity index significantly increases with increasing wealth status, with no significant difference between altitudinal gradients. The finding indicates that household economic status determines the diversification of woody species in ecologically related human-managed land use systems. Therefore, to improve woody species diversification in homegarden agroforestry, concerned bodies should support the residents through the provision of appropriate supplies. Furthermore, more investigation will be required to determine the specific variables influencing the woody species composition and diversity in homegarden agroforestry and its sustainable socio-economic and ecological uses.

References

1. Bishaw, B & Abdelkadir, A. (2003). Agroforestry and Community Forestry for Rehabilitation of Degraded Watersheds on the Ethiopian Highlands. International Conference on African Development Archives. 78. https://scholarworks.wmich.edu/africancentericad-archive/78.
2. Sistla, S.A., Roddy, A.B., Williams, N.E., Kramer, D.B., Stevens, K & Allison, S.D. (2016). Agroforestry Practices Promote Biodiversity and Natural Resource Diversity in Atlantic Nicaragua. *PLoSONE,* **11**(9).

3. Montagnini, F. (2020). The Contribution of Agroforestry to Restoration and Conservation: Biodiversity Islands in Degraded Landscapes. In: J. C. Dagar *et al*. (eds.), Agroforestry for Degraded Landscapes: Recent Advances and Emerging Challenges - Vol. 1, https://doi.org/10.1007/978-981-15-4136-0_15
4. Nair, P.K.R. (1993). An Introduction to Agroforestry. Kluwer Academic Publishers-ICRAF. P. 489.
5. Harvey, C.A & Villalobos, J.A.G. (2007). Agroforestry systems conserve species-rich but modified assemblages of tropical birds and bats. *Biodiversity and Conservation*, **16**: 2257-2292.
6. Bardhan, S., Jose, S., Biswas, S., Kabir, K & Rogers, W. (2012). Homegarden agroforestry systems: an intermediary for biodiversity conservation in Bangladesh. *Agroforest Syst*, **85**:29–34.
7. Jama, B., Elias, E & Mogotsi, K. (2006). Role of agroforestry in improving food security and natural resource management in the drylands: a regional overview. *Journal of the Drylands*, **1**(2): 206-211.
8. Bucheli, V.J.P & Bokelmann, W. (2017). Agroforestry systems for biodiversity and ecosystem services: the case of the Sibundoy Valley in the Colombian province of Putumayo. *International Journal of Biodiversity Science, Ecosystem Services & Management*, **13**(1): 380-397.
9. Nair, P.K.R. (2017). Managed Multi-strata Tree + Crop Systems: An Agroecological Marvel. *Front. Environ. Sci*. **5**:88. doi: 10.3389/fenvs.2017.00088.

10. Rendón-Sandoval, F.J., Casas, A., Moreno-Calles, A.I., Torres-García, I & García-Frapolli, E. (2020). Traditional Agroforestry Systems and Conservation of Native Plant Diversity of Seasonally Dry Tropical Forests. *Sustainability,* **12**: 4600; doi:10.3390/su12114600.

11. Reang, D., Hazarika, A., Sileshi, G.W., Pandey, R., Das, A.K & Nath, A.J. (2021). Assessing tree diversity and carbon storage during land use transitioning from shifting cultivation to indigenous agroforestry systems: Implications for REDD+ initiatives. *Journal of Environmental Management*, **298**: 113470.

12. Endale, Y., Derero, A., Argaw, M & Muthuri, C. (2017). Farmland tree species diversity and spatial distribution pattern in semi-arid East Shewa, Ethiopia. *Forests, Trees and Livelihoods*, **26**(3): 199-214. https://doi.org/10.1080/14728028.2016.1266971

13. van Noordwijk, M., Rahayu, S., Gebrekirstos, A., Kindt, R., Tata, H.L., Muchugi, A *et al*. (2019). Tree diversity as basis of agroforestry. In: van Noordwijk M, ed. Sustainable development through trees on farms: agroforestry in its fifth decade. Bogor, Indonesia: World Agroforestry (ICRAF) Southeast Asia Regional Program. pp 17−44.

14. Tadesse, E., Negash, M & Asfaw, Z. (2021). Impacts of traditional agroforestry practices, altitudinal gradients and households' wealth status on perennial plants species composition, diversity, and structure in south-central Ethiopia. *Agroforestry Systems*, ***95***: 1533-1561.

15. Abebe, T. (2005). Diversity in homegarden agroforestry system of southern Ethiopia. PhD Dissertation, Wageningen Agricultural University. The Netherlands.

16. Beetz, A.E. (2011). Agroforestry: An Overview. The National Sustainable Agriculture Information Service, ATTRA, http://www.attra.ncat.org

17. Leakey, R.R.B. (2017). Definition of agroforestry revisited. In: Multifunctional Agriculture – Achieving Sustainable Development in Africa, RRB Leakey, 5-6, Academic Press, San Diego, California, USA.

18. Korwar, G.R., Prasad, J.V.N.S., Rajeshwara Rao, G., Venkatesh, G., Pratibha, G & Venkateswarlu, B. (2018). Agroforestry as a Strategy for Livelihood Security in the Rainfed Areas: Experience and Expectations. In J.C. Dagar *et al*. (eds.). Agroforestry Systems in India: Livelihood Security & Ecosystem Services. *Advances in Agroforestry*, **10**. https://10.1007/978-81-322-1662-9_5

19. Dawson, I.K., Guariguata, M.R., Loo, J., Weber, J.C., Lengkeek, A., Bush, D *et al*. (2013). What is the relevance of smallholders' agroforestry systems for conserving tropical tree species and genetic diversity in circa situm, in situ and ex situ settings? A review. *Biodivers Conserv*, **22**(2): 301–324.

20. Schroth G., Fonseca, G.A.B., Harvey, C.A., Gascon, C., Vasconcelos, H.L & Izac, A.M.N. (2004). Agroforestry and Biodiversity Conservation in Tropical Landscapes. Island Press, Washington.

21. Jose, S. (2009). Agroforestry for ecosystem services and environmental benefits: an overview. *Agroforestry Systems*, **76**:1–10.

22. Montagnini, F., Francesconi, W & Rossi, E. (ed.) (2011). Agroforestry as a tool for landscape restoration. Nova Science, New York.

23. Torquebiau, E & Penot, E. (2006). Ecology versus economics in tropical multistrata agroforests. In: B.M. Kumar & P.K.R. Nair (eds). Tropical homegardens: A time-tested example of sustainable agroforestry, 269-282.

24. Mohan, S. (2004). An assessment of the ecological and socioeconomic benefits provided by homegardens: a case study of Kerala, India. University of Florida.

25. Gebremedhin, T., & Mulubrehan, H. (2007). Women in backyards: root crop production and biodiversity management in backyards. A case study in five selected woredas of tigray regional state, Northern Ethiopia. Northern Ethiopia DCG Report No, 50.

26. Maroyi, A. (2009). Traditional homegardens and rural livelihoods in Nhema, Zimbabwe: a sustainable agroforestry system. *International Journal of Sustainable Development & World Ecology*, **16**(1): 1-8.

27. Park, J.H., Woo, S.Y., Kwak, M.J., Lee, J.K., Leti, S & Soni, T. (2019). Assessment of the Diverse Roles of Home Gardens and Their Sustainable Management for Livelihood Improvement in West Java, Indonesia. *Forests*, **10**(970).

28. Eyasu, G., Tolera, M & Negash, M. (2020). Woody species composition, structure, and diversity of homegarden agroforestry systems in southern Tigray, Northern Ethiopia. *Heliyon*, **6**(12), e05500.

29. Kassa, G., Bekele, T., Demissew, S & Abebe, T. (2023). Plant species diversity, plant use, and classification of agroforestry homegardens in southern and southwestern Ethiopia. *Heliyon*, **9**.

30. George, M. V & Christopher, G. (2020). Structure, diversity and utilization of plant species in tribal homegardens of Kerala, India. *Agroforestry systems*, ***94***(1): 297-307.

31. Sood, K.K & Mitchell, C.P. (2009). Identifying important biophysical and social determinants of on-farm tree growing in subsistence-based traditional agroforestry systems. *Agroforest Syst*, **75**:175–187.

32. Bayala, J., Kindt, R., Belem, M & Kalinganire, A. (2011). Factors affecting the dynamics of tree diversity in agroforestry parklands of cereal and cotton farming systems in Burkina Faso. *New Forests*, **41**:281–296.

33. Nyaga, J., Barrios, E., Muthuri, C.W., Öborn, I., Matiru, V & Sinclair, F.L. (2015). Evaluating factors influencing heterogeneity in agroforestry adoption and practices within smallholder farms in Rift Valley, Kenya. *Agriculture, Ecosystems and Environment*, **212**: 106–118.

34. Guyassa, E & Raj, A.J. (2013). Assessment of biodiversity in cropland agroforestry and its role in livelihood development in dryland areas: A case study from Tigray region, Ethiopia. *International Journal of Agricultural Technology*, **9**(4): 829-844.

35. Mekonnen, E.L., Asfaw, Z & Zewudie, S. (2014). Plant species diversity of homegarden agroforestry in Jabithenan District, North-Western Ethiopia. *Int. J. Biodivers. Conserv.* ***6***(4): 301-307.

36. Tadesse, E., Abdulkedir, A., Khamzina, A., Son, Y & Noulèkoun, F. (2019). Contrasting Species Diversity and Values in Home Gardens and Traditional Parkland Agroforestry Systems in Ethiopian Sub-Humid Lowlands. *Forests*, **10**(266).

37. Asfaw, B & Lemenih, M. (2010). Traditional agroforestry systems as a safe haven for woody plant species: a case study from a topo-climatic gradient in South Central Ethiopia. *Forests, Trees and Livelihoods*, ***19***(4): 359-377.

38. Gebrewahid, Y & Abrehe, S. (2019). Biodiversity conservation through indigenous agricultural practices: Woody species composition, density and diversity along an altitudinal gradient of Northern Ethiopia. *Cogent Food & Agriculture,* **5**:1.

39. Wari, B.N., Feyssa, D.H & Kebebew, Z. (2019). Assessment of woody species in agroforestry systems around Jimma Town, Southwestern Ethiopia. *International Journal of Biodiversity and Conservation,* **11**(1): 18-30.

40. Henry, M., Tittonell, P., Manlay, R.J., Bernoux, M., Albrecht, A & Vanlauwe, B. (2009). Biodiversity, carbon stocks and sequestration potential in aboveground biomass in smallholder farming systems of western Kenya. *Agriculture, ecosystems & environment*, ***129***(1-3): 238-252.

41. Legesse, A & Negash, M. (2021). Species diversity, composition, structure and management in agroforestry systems: the case of Kachabira district, Southern Ethiopia. *Heliyon*, **7**(3): e06477.

42. Motuma, T., Zebene, A., Mulugeta, L & Karltun, E. (2008). Woody species diversity in a changing landscape in the south-central highlands of Ethiopia. *Agriculture, Ecosystems and Environment*, **128**: 52–58.

43. Azene B., Birnie, A & Tengnäs, B. (1993). Useful trees and shrubs for Ethiopia: Identification, propagation and management for agricultural and pastoral communities. SIDA's regional Soil Conservation Unit. Ethiopia. P. 474.

44. Edwards, S., Tadesse, M & Hedberg, I. (1995). Flora of Ethiopia and Eritrea, Vol. 2, Part 2, The National Herbarium, Addis Ababa University, Addis Ababa, Ethiopia and Department of Systematic Botany, Uppsala University, Uppsala, Sweden.

45. Hedberg, I & Edwards, S. (1995). Flora of Ethiopia and Eritrea Vol. 7. Poaceae. The National Herbarium, Addis Ababa, Ethiopia, and Department of Systematic Botany, Uppsala, Sweden.

46. Hedberg, I., Kelbessa, E., Edwards, S., Demissew, S & Persson, E. (2006). Flora of Ethiopia and Eritrea Vol. 5. Gentianaceae to Cyclocheilaceae. The National Herbarium, Addis Ababa University, Addis Ababa, Ethiopia and Department of Systematic Botany, Uppsala University, Uppsala, Sweden.

47. Krebs, C.J. (1989). Ecological methodology. New York: Harper Collins Publishers.

48. Kent, M & Coker, P. (1992). Vegetation description and analysis. A practical approach. London: Belhaven Press.

49. Magurran, A.E. (2004). Measuring biological diversity. Malden and Oxford: Blackwell Publishing.

50. R Core Team. (2021). R: A language and environment for statistical computing. R Foundation for Statistical Computing, Vienna, Austria. URL, https://www.R-project.org/.

51. Molla, T., Asfaw, A., Muluneh, M.G & Worku, B.B. (2023). Diversity of woody species in traditional agroforestry practices in Wondo district, south-central Ethiopia. *Heliyon*, **9** e13549. https://doi.org/10.1016/j.heliyon.2023.e13549

52. Tesfay, F., Moges, Y & Asfaw, Z. (2022). Woody species composition, structure, and carbon stock of coffee-based agroforestry system along an elevation gradient in the moist mid-highlands of Southern Ethiopia. *International Journal of Forestry Research, 2022*.

53. Molla, A & Kewessa, G. (2015). Woody Species Diversity in Traditional Agroforestry Practices of Dellomenna District, Southeastern Ethiopia: Implication for Maintaining Native Woody Species. *International Journal of Biodiversity*, **2015**, 13

54. Cardozo, E.G., Muchavisoy, H.M., Silva, H.R., Zelarayán, M.L.C., Leite, M.F.A., Rousseau, G.X *et al*. (2015). Species richness increases income in agroforestry systems of eastern Amazonia. *Agroforestry systems*, **89**: 901-916.

55. Pandey, H.P., Pokhrel, N.P., Luitel, D.R., Acharya, K & Shah, K.K. (2021). Diversity of agroforestry species and uses in two ecological regions: A Case from Central Nepal. *Advances in Agriculture*, ***2021***: 1-9.

56. Woldeamanual, W., Asfaw, Z & Lemessa, D. (2018). The Efect of the Surrounding Landscape and Socioeconomic characteristics on Woody Species Diversity in Homegardens of Shewarobit District, Northeast Ethiopia. *Small-scale Forestry*, https://doi.org/10.1007/s11842-018-9398-1

57. Jegora, T., Asfaw, Z & Anjulo, A. (2019). Woody Species Diversity and Management in Homegarden Agroforestry: The Case of Shashemene District, Ethiopia. *International Journal of Forestry Research*, **2019**, 3697047.

58. Birhane, E., Ahmed, S., Hailemariam, M., Negash, M., Rannestad, M.M & Norgrove, L. (2020). Carbon stock and woody species diversity in homegarden agroforestry along an elevation gradient in southern Ethiopia. *Agroforest Syst*, https://doi.org/10.1007/s10457-019-00475-4.

59. Temgoua, L.F., Etchike, A.B.D., Solefack, M.C.M., Tumenta, P & Nkwelle, J. (2020). Woody species diversity conservation and carbon sequestration potential of coffee agroforestry systems in the Western Region of Cameroon. *Journal of Horticulture and Forestry*, **12**(2): 35-48.

60. Bajigo, A & Tadesse, M. (2015). Woody species diversity of traditional agroforestry practices in Gununo watershed in Wolayitta zone, Ethiopia. *Forest Research*, **4**(4): 2168-9776.

61. Chemeda, B.A., Wakjira, F.S & Hizikias, E.B. (2022). Tree diversity and biomass carbon stock analysis along altitudinal gradients in coffee based agroforestry system of Western Ethiopia. *Cogent Food & Agriculture*, **8**(1): 2123767.

62. Gebrehiwot, M., Elbakidze, M., Lidestav, G., Sandewall, M., Angelstam, P & Kassa, H. (2016). From self-subsistence farm production to khat: driving forces of change in Ethiopian agroforestry homegardens. *Environmental Conservation*, **43**(3): 263-272.

Appendix 1: List of woody species scientific, local and family name, and their origin in homegarden agroforestry systems of southwest Ethiopia

**№**	**Scientific name**	**Local name**	**Family name**	**Origin**
1	*Acacia abyssinica* Hochst. ex Benth.	Sondii	Fabaceae	I
2	*Albizia gummifera* (J. F. Gmel.) C.A. Sm.	Ambabbessa	Fabaceae	I
3	*Albizia schimperiana* Oliv.	Ambabbessa arba	Fabaceae	I
4	*Allophylus abyssinicus* (Hochst.) Radikofer	Sehoo	Sapindaceae	I
5	*Annona senegalensis* pers.	Gishxaa	Annonaceae	I
6	*Artocarpus heterophyllus*	Jakifiruttii	Moraceae	E

7	*Azadirachta indica* A.Juss.	Niimii	Meliaceae	E
8	*Borassus aethiopum* Mart.	Meexxii	Arecaceae	I
9	*Brucea antidysenterica* J.F.Miller	Qomonyo	Simaroubaceae	I
10	*Calpurnia aurea* (Ait.) Benth.	Ceekaa	Fabaceae	E
11	*Carica papaya* L.	Papayee	Caricaceae	I
12	*Casimiroa edulis* LaLava	Kashimirii	Rutaceae	E
13	*Casuarina equisetifolia*	Shuwashuwe	Casuarinaceae	E
14	*Catha edulis* (Vahl) Forssk. ex Endl.	Caatii	Celastraceae	I
15	*Citrus aurantifolia* (Christm.) Swingle	Loomii	Rutaceae	E
16	*Citrus medica*	Marara	Rutaceae	E
17	*Citrus sinensis*	Burtukana	Rutaceae	E
28	*Coffea arabica* L	Buna	Rubiaceae	I
19	*Cordia africana* Lam.	Waddeessa	Boraginaceae	I
20	*Croton macrostachyus* Hochst ex Del.	Bakkannisa	Euphorbiaceae	I
21	*Cupressus lusitanica*	Gattira faranjii	Cupressaceae	E
22	*Dovyalis abyssinica* (A. Rich.) Warb.	Koshimii	Flacourtiaceae	I

23	*Dracaena fragrans*	Omo	Dracaenaceae	I
24	*Dracaena steudneri* Engl.	Bubbiftu	Dracaenaceae	I
25	*Duranta erecta* L.	Ababa	Verbenaceae	E
26	*Ehretia cymosa* Thonn.	Ulaaga	Boraginaceae	I
27	*Erythrina abyssinica* Lam. ex DC.	Waleensuu	Fabaceae	I
28	*Erythrina brucei* Schweinf.	Bero	Fabaceae	I®
29	*Eucalyptus camaldulensis*	Bargamo diimaa	Myrtaceae	E
30	*Eucalyptus citriodora* Hook.	Bargamo	Myrtaceae	E
31	*Eucalyptus globules* Labill.	Bargamo adii	Myrtaceae	E
32	*Ficus thonningii* Blume	Dambii	Moraceae	I
33	*Ficus sur* Forssk.	Harbuu	Moraceae	I
34	*Grevillea robusta* A.Cunn. ex R.Br.	Giravillaa	Proteaceae	E
35	*Jacaranda mimosifolia* D.Don	Jakaranda	Bignoniaceae	E
36	*Maesa lanceolata* Forssk.	Abbayyii	Myrsinaceae	I
37	*Malus domestica*	Appilii	Rosaceae	E
38	*Mangifera indica* L.	Mango	Anacardiaceae	E

39	*Millettia ferruginea* (Hochst.) Baker	Askira	Fabaceae	I®
40	*Moringa oleifera*	Moriinga	Moringaceae	E
41	*Morus alba* L.	Goraa	Moraceae	E
42	*Ocimum lamiifolium* Hochst. ex Benth	Damakase	Lamiaceae	I
43	*Olea europaea* (Wall. ex DC.) Cifferri	Ejersa	Oleaceae	I
44	*Olea welwitschii* (Knobl.) Gilg & Schellenb.	Baya	Oleaceae	I
45	*Persea americana* Mill.	Avokado	Lauraceae	E
46	*Pouteria campechiana*	Eggifiruttii	Sapotaceae	E
47	*Prunus persica* (L.) Batsch	Kukko	Rosaceae	E
48	*Psidium guajava* L.	Zetuna	Myrtaceae	E
49	*Rhamnus prinoides* L'Herit	Gesho	Rhamnaceae	I
50	*Ricinus communis* L.	Qobbo	Euphorbiaceae	I
51	*Sapium ellipticum* (Hochst) Pax.	Bosoqa	Euphorbiaceae	I
52	*Sesbania sesban*	Sasbaniya	Fabaceae	I
53	*Spathodea campanulata*	Ispatoda	Bignoniaceae	E
54	*Vernonia amygdalina* Del.	Eebicha	Asteraceae	I

55	*Vernonia auriculifera* Heirn.	Reejjii	Asteraceae	I

Key: Origin: I- Indigenous, E- Exotic, ®- Endemic species

Appendix 2: Percentage frequency (%Freq), relative dominance (Rdo), relative density (Rde), relative frequency (RF) and importance value index (IVI) of woody species across the entire homegarden agroforestry systetms

**N/S**	**Scientific Name**	**% Freq**	**Rdo**	**Rde**	**RF**	**IVI**
1	*Catha edulis*	54.2	7.4	54.5	7.1	69.1
2	*Coffea arabica*	84.7	10.6	29.3	11.1	51.0
3	*Persea americana*	79.2	27.5	3.0	10.4	40.8
4	*Cordia africana*	44.4	11.2	1.1	5.8	18.1
5	*Mangifera indica*	44.4	9.0	1.0	5.8	15.8
6	*Grevillea robusta*	36.1	6.3	1.0	4.7	12.0
7	*Psidium guajava*	37.5	3.8	0.6	4.9	9.3
8	*Citrus sinensis*	34.7	3.9	0.7	4.6	9.2
9	*Ricinus communis*	40.3	1.2	1.5	5.3	8.0
10	*Carica papaya*	20.8	3.7	0.5	2.7	6.9

11	*Albizia gummifera*	27.8	2.4	0.4	3.6	6.4
12	*Vernonia auriculifera*	30.6	0.0	1.0	4.0	5.0
13	*Cupressus lusitanica*	18.1	1.2	0.7	2.4	4.3
14	*Annona senegalensis*	18.1	1.3	0.5	2.4	4.1
15	*Duranta erecta*	23.6	0.0	0.7	3.1	3.8
16	*Croton macrostachyus*	13.9	0.8	0.3	1.8	2.9
17	*Erythrina brucei*	11.1	0.8	0.3	1.5	2.6
28	*Vernonia amygdalina*	12.5	0.7	0.1	1.6	2.4
19	*Jacaranda mimosifolia*	6.9	1.3	0.1	0.9	2.3
20	*Casimiroa edulis*	11.1	0.7	0.2	1.5	2.4
21	*Sesbania sesban*	12.5	0.1	0.4	1.6	2.2
22	*Albizia schimperiana*	6.9	0.8	0.1	0.9	1.8
23	*Millettia ferruginea*	4.2	1.1	0.1	0.6	1.7
24	*Calpurnia aurea*	9.7	0.1	0.4	1.3	1.7
25	*Rhamnus prinoides*	6.9	0.0	0.2	0.9	1.1
26	*Ehretia cymosa*	4.2	0.4	0.1	0.6	1.0

27	*Acacia abyssinica*	1.4	0.7	0.1	0.2	0.9
28	*Malus domestica*	5.6	0.0	0.3	0.7	1.0
29	*Erythrina abyssinica*	5.6	0.1	0.1	0.7	0.9
30	*Citrus aurantifolia*	5.6	0.1	0.1	0.7	0.9
31	*Olea europaea*	4.2	0.2	0.1	0.6	0.8
32	*Casuarina equisetifolia*	2.8	0.4	0.0	0.4	0.8
33	*Eucalyptus citriodora*	2.8	0.2	0.1	0.4	0.7
34	*Eucalyptus globulus*	2.8	0.2	0.1	0.4	0.6
35	*Dovyalis abyssinica*	2.8	0.1	0.1	0.4	0.5
36	*Azadirachta indica*	2.8	0.1	0.0	0.4	0.5
37	*Olea welwitschii*	2.8	0.1	0.0	0.4	0.5
38	*Citrus medica*	2.8	0.1	0.0	0.4	0.5
39	*Dracaena steudneri*	1.4	0.3	0.0	0.2	0.5
40	*Artocarpus heterophyllus*	6.9	0.2	0.0	0.2	0.5
41	*Maesa lanceolata*	2.8	0.1	0.0	0.4	0.5
42	*Moringa oleifera*	1.4	0.2	0.0	0.2	0.4

43	*Morus alba*	2.8	0.0	0.0	0.4	0.4
44	*Borassus aethiopum*	1.4	0.2	0.0	0.2	0.4
45	*Allophylus abyssinicus*	1.4	0.2	0.0	0.2	0.4
46	*Pouteria campechiana*	1.4	0.1	0.0	0.2	0.3
47	*Ficus sur*	1.4	0.1	0.0	0.2	0.3
48	*Dracaena fragrans*	1.4	0.1	0.1	0.2	0.3
49	*Ficus thonningii*	1.4	0.1	0.0	0.2	0.3
50	*Eucalyptus camaldulensis*	1.4	0.1	0.0	0.2	0.3
51	*Prunus persica*	1.4	0.1	0.0	0.2	0.3
52	*Spathodea campanulata*	1.4	0.1	0.0	0.2	0.3
53	*Sapium ellipticum*	1.4	0.1	0.0	0.2	0.3
54	*Ocimum lamiifolium*	1.4	0.0	0.0	0.2	0.2
55	*Brucea antidysenterica*	1.4	0.0	0.0	0.2	0.2

Manuscript

Click here to access/download;Manuscript;Teferas diversity final.pdf

Woody species composition and diversity of homegarden agroforestry along altitudinal gradient in southwest Ethiopia

Tefera Jegora[1, 2,], Kitessa Hundera[1], Zerihun Kebebew[1] and Adugna Eneyew[1]*

*[1]College of Agriculture and Veterinary Medicine, Jimma University, Ethiopia; [2]College of Agriculture and Forestry, Mettu University, Ethiopia, *- Corresponding author e-mail: teferajegora@gmail.com*

Abstract

Homegarden agroforestry systems that integrate trees with agricultural practices are usually known for the conservation of farm biodiversity. Despite the system having a significant conservation role, limited information was revealed on woody species composition and diversity following the elevation belt of southwest Ethiopia. A complete enumeration of 72 homegardens (24 each from altitudinal gradient) was purposively selected for woody species inventory. A total of 55 woody species belonging to 31 families and 45 genera were recorded. Of which, 56.4% of woody species are indigenous and two are endemic to Ethiopia. Family Fabaceae is the most dominant woody having eight species. The highest species richness (42) was observed at high altitudes followed by 39 species at middle and 31 species at low altitudes but no significant difference between them. Species richness significantly ($P < 0.001$) increased with increasing wealth status. The overall richness distribution was 46, 40, and 27 across rich, medium, and poor wealth classes respectively. Shannon diversity index was significant between poor and rich households. Pearson correlation result shows a strong positive and significant correlation between richness and wealth status. The mean woody species density was (89.06±9.25) and (1236.22±131.42) per garden and hectare respectively. Stem density showed a significant

difference (P < 0.001) between wealth statuses. Therefore, the household economy significantly determines woody species composition and diversity of homegarden agroforestry systems in southwest Ethiopia.

Key words: *Altitudinal change, species richness, wealth status, woody diversity*

1. BACKGROUND

Agroforestry system is a strategic land use plan to conserve biodiversity in deforested and fragmented landscapes (Bishaw & Abdelkadir, 2003; Sistla *et al*., 2016; Montagnini, 2020). The practice integrates the most environmentally sound forestry and agricultural systems into the same land management unit with sustainable and profitable interaction between trees-crops-livestock (Nair, 1993). It supports the floral and faunal assemblages that can be as species-rich, abundant, and diverse as forests with modified and non-forest species compositions (Harvey & Villalobos, 2007; Bardhan *et al*., 2012). These help agroforestry systems to have diverse components and multi-strata structures having multiple ecological and socio-economic benefits (Jama *et al*., 2006; Bucheli & Bokelmann, 2017; Nair, 2017).

Agroforestry is a potential biodiversity conservation site following the intact natural forests as it provides important reservoirs for terrestrial biodiversity (Rendón-Sandoval *et al*., 2020; Reang *et al*., 2021). Tropical agroforestry systems have high levels of woody species diversity which are naturally retained and/ or planted on farmland (Endale *et al*., 2017; van Noordwijk *et al*., 2019; Tadesse *et al*., 2021). The system encourages tree plantation and maintenance to diversify multipurpose woody species as a means of smallholder farmer's livelihood (Beetz, 2011; Leakey, 2017; Korwar *et al*., 2018). Agroforestry systems serve as *in situ* conservations containing high species richness and diversity valued and conserved by the farmers (Dawson *et al*., 2013; Rendón-Sandoval *et al*., 2020). As a result, agroforestry plays five vital roles in

biodiversity conservation: (1) provides habitat for species to tolerate a certain level of disturbance; (2) reduce rate of conversion of natural habitat by providing a more productive and sustainable alternative agricultural systems; (3) provides corridors between habitat remnants and the conservation of area-sensitive floral and faunal species; (4) preserve germplasm of sensitive species; and (5) providing ecosystem services such as erosion control, increase infiltration capacity, reduce degradation and habitat fragmentation (Schroth *et al*., 2004; Jose, 2009; Montagnini *et al*., 2011; Montagnini, 2020).

Homegarden agroforestry is usually managed by the members of households for the production of subsistence crops and sometimes cash objectives (Torquebiau & Penot, 2006). The diverse and multi-strata components of the system not only provide significant food and income sources (Mohan, 2004; Gebremedhin & Mulubrehan, 2007; Maroyi, 2009; Park *et al*., 2019) but also biodiversity conservation (Eyasu *et al*., 2020; Kassa *et al*., 2023). Constantly, it varies from the nearby agroforestry practices due to their mosaic patches or plots dominated by a diversity of woody species (Abebe *et al*., 2010). Having multiple strata, complex structure, high species richness, evenness, and abundance make homegardens typically similar to natural forests (Abebe, 2005; Bardhan *et al*., 2012; George & Christopher, 2019). Homegarden contains high species diversity and usually 3-4 vertical canopy strata including trees as an upper storey, herbaceous as near ground, and intermediate layers in between (Nair, 1993; Abebe, 2005; Jama *et al*., 2006). This composite nature of the agroforestry system is under the influence of different ecological and socio-economic factors globally (Sood & Mitchell, 2009; Bayala *et al*., 2011; Tadesse *et al*., 2021). For example: Nyaga *et al*. (2015) revealed that household level of resource endowment significantly affects tree number, species richness, and species diversity.

In Ethiopia, agroforestry is an old farming system containing low to high woody species diversity distribution due to variations in ecological and socio-economic factors such as elevation, size of farmland, and wealth status (Abebe, 2005, Gebremedhin & Mulubrehan, 2007; Guyassa & Raj, 2013; Mekonnen *et al*., 2014; Tadesse *et al*., 2019). According to Asfaw & Lemenih (2010), woody species composition in the agroforestry system is influenced by household wealth status and other socio-economic attributes such as size of farmland, level of education, family size, and off-farm economic sources. In indigenous agricultural practices of the northern Ethiopian homegardens, Gebrewahid & Abrehe (2019) reported variations between woody species composition, density, and diversity along altitudinal gradients. Additionally, there is a recommendation by Wari *et al*. (2019) for further investigation into the interaction between homegarden agroforestry woody species and their physical environment in southwestern Ethiopia. Therefore, this study was conducted to deal with woody species composition and diversity of homegarden agroforestry systems along an altitudinal gradient and household wealth status in southwest Ethiopia.

2. MATERIALS AND METHODS

2.1. Description of the Study Area

The study was conducted in three districts namely: Mana, Gomma and Dhidhessa following the elevation belt of southwest Ethiopia. The area is located between 7°40ˈN-8°10ˈN and 36°30ˈE-36°50ˈE with an elevation range between 1459m to 2542m above sea level (Fig. 1). The area received a mean annual rainfall of 1570mm with 13-28°C minimum and maximum temperature respectively. It is occupied by cultivated land, wetlands, settlements, commercial farms, shrubland, grassland, and forest land. Agro-ecologically, it is classified into lowland, midland and highland with midland-dominated areas. Agriculture is the main source of socio-economic

development having coffee and khat as dominant cash crops. Homegarden agroforestry (coffee, ensent and khat) based system is the most accredited farming systems.

2.2. Sampling Strategy

Woody species inventory was conducted in homegarden agroforestry systems across the elevation belt of southwest Ethiopia. Elevation classification was made purposively based on the presence of homegarden agroforestry systems. Elevation data was gathered from the Digital Elevation Model in Google Earth (Henry *et al*., 2009). Then, three altitudinal gradients were made as low (1459-1750m), middle (1750-2050m) and high (2050-2542m) following the distributions of homegarden agroforestry with its perspective altitude to see the effect of altitudinal change on woody species diversity. Additionally, wealth status was used as it positively or negatively affects woody species in homegarden agroforestry systems and classified as rich, medium and poor (Legesse & Negash, 2021; Tadesse *et al*., 2021). The classification was made with the assistance of the developmental agents and local elders. Here, the developmental agent refers to agricultural extesion worker at the smallest admistrative unit (locally called kebele) in Ethiopia. Local criteria such as the number of livestock holding, total size of land holding, housing standard and annual consumption food and sold crop production were used for this classification. Then, a complete homegarden enumeration was made for the woody species inventory (Motuma *et al*., 2008). Finally, a total of 72 homegardens (24 from each altitudinal gradient) were purposively selected for woody species inventory.

2.3. Methods of Data Collection

Woody species with a diameter at breast height (DBH) ≥5cm and height ≥1.5m were identified and their height and DBH were measured. Individuals below this measurement were only

counted and registered. For woody branched below 1.3m above the ground, the average DBH was calculated from all branches measured separately (Tadesse *et al*., 2019). Tree DBH and height were measured using a caliper and Sunto Clinometer respectively. Tree size beyond the caliper measurement was measured using mater tape and converted to diameter. Tree height difficult to measure with a Sunto Clinometer was measured by a marked range pole. The altitudinal gradient was measured using the Global Positioning System (GPS) Garmin 72. Woody species identification was made on the field and voucher specimen. A voucher specimen was collected, pressed, dried and identified following Azene *et al.* (1993) and flora of Ethiopia (Edwards *et al*., 1995; Hedberg & Edwards, 1995; Hedberg *et al*., 2003; 2006).

2.4. Data Analysis

Woody species diversity was analyzed using diversity indices (Krebs, 1989; Kent and Coker, 1992; Magurran, 2004). Shannon Wiener diversity index which takes species richness and evenness into consideration was calculated as:

$$H' = -\sum_{i=1}^{s} pilnpi \ldots\ldots\ldots\ldots\ldots\ldots\ldots\ldots\ldots\ldots\ldots\ldots\ldots\ldots\ldots\ldots\ldots\ldots \quad (1)$$

Where: H' = Shannon-Wiener diversity index, s = number of species and Pi = the proportion of individuals of the ith species and ln =s natural logarithm.

A measure of similarity of the abundances of different woody species in the sampled area was analyzed using Evenness/Equitability index.

$$E = \frac{H'}{Hmax} = \frac{H'}{lnS} \; with\, Hmax = lnS \ldots\ldots\ldots\ldots\ldots\ldots\ldots\ldots\ldots\ldots\ldots\ldots\ldots \quad (2)$$

Where: E = Evenness (Equitability), Hmax = ln(S) is the maximum level of diversity possible within a given population.

The similarity in species composition of the two sites is computed using Sorensen's Similarity Coefficient (S_s) with values ranging between 0 and 1: 0 indicates complete dissimilarity and 1 complete similarity.

$$Ss = \frac{2a}{2a+b+c} \ldots\ldots\ldots\ldots\ldots\ldots\ldots\ldots\ldots\ldots\ldots\ldots\ldots\ldots\ldots\ldots \quad (3)$$

Where: Ss = Similarity index, a = number of species common in the two sites; b = number of species recorded only in site one; and c = number of species recorded only in site two.

Importance value index (IVI) is a useful ecological parameter to compare the environmental significance of woody species (Lamprecht, 1989). IVI was calculated using the sum of relative density, frequency and dominance as:

IVI= Relative density + Relative dominance + Relative frequency ……………………… *(4)*

Where: $\text{Relative Density (RDe)} = \frac{\text{Stem count of a species}}{\text{Stem count of all species}} * 100 \ldots\ldots\ldots\ldots\ldots\ldots 4.1$

$$\text{Relative Dominance (RDo)} = \frac{\text{Doninance of a species}}{\text{Dominance of all species}} * 100 \ldots\ldots\ldots\ldots\ldots 4.2$$

$$\text{Relative Frequency (RF)} = \frac{\text{Frequency of a species}}{\text{Frequency of all species}} * 100 \ldots\ldots\ldots\ldots\ldots 4.3$$

The density of woody species was calculated by converting the total number of individuals encountered in all plots to equivalent number per hectare using the following formula:

$$D = N/S \ldots\ldots\ldots\ldots\ldots\ldots\ldots\ldots\ldots\ldots\ldots\ldots\ldots\ldots\ldots\ldots \quad (5)$$

Where: D - the density (stems ha^{-1}), N - the number of stems present on the considered surface and S - the area considered in hectare (ha).

Basal area, the area occuied by woody species was calculated for all woody species DBH ≥5cm using the following formula:

Basal area (BA) = $\pi d^2/4$ ……………………………………………………………………………… *(6)*

Where: π= 3.14; d = diameter at breast height (m)

Statistical analysis was made using R software version 4.1.3 (R Core Team, 2021). Homogeneity and normality of the variance were approved using the LeveneTest and Shapiro-Wilk test in R statistics. Non-normally distributed data was analyzed using log transform and removal of the outlier. The variables were compared using one-way ANOVA (analysis of variance). The relationship between altitudinal gradients and wealth status with the diversity indices was seen using Pearson's correlation coefficient test. The Least Significance Difference (LSD) test was used to compare the level of significance ($P < 0.05$) between each variable.

3. RESULTS

3.1. Floristic composition

A total of 55 woody species belonging to 31 families and 45 genera were recorded in homegarden agroforestry systems. Family *Fabaceae* is the most dominant having eight species followed by *Moraceae, Myrtaceae* and *Rutaceae* each with four species. About 34.5% of the remaining families had only a single woody species. About 56.4% of species are indigenous and 43.6% exotic including two endemics (*Erythrina brucei* and *Millettia ferruginea*) (Appendix, 1). The total species richness was 31, 39 and 42 for low, middle and high altitudes respectively. Richness increases with wealth status as 27 for poor, 40 for medium and 46 for rich households (Table 1). Species richness was not significant ($P < 0.05$) between altitudinal gradients whereas it significantly ($P < 0.001$) increased with increasing wealth status (Table 2). The percentage frequency occurrence of the study area woody species lay between 1.4 - 84.7%. *Coffea arabica* (84.7%), *Persea americana* (79.5%), and *Catha edulis* (54.2%) are the most frequenstly recorded

woody species. However, about 15 woody species observed only once in surveyed homegarden agroforestry systems (Appendix 2).

Table 1: Overall woody species richness, genera and family distiribution of woody species across altitudinal gradient and wealth status in homegarden agroforestry systems

**Category**	**Number**	**Richness**	**Genus**	**Family**
**Altitudinal gradient**				
Low	24	31	26	21
Middle	24	39	36	25
High	24	42	37	25
Total	***72***	***55***	***45***	***31***
Wealth class				
Poor	*24*	*27*	*24*	*21*
Medium	*24*	*40*	*33*	*24*
Rich	*24*	*46*	*39*	*27*
Total	***72***	***55***	***45***	***31***

Table 2: Species richness, Shannon diversity index and Evenness (Mean ± SE) of woody species across altitudinal gradients and wealth status in southwest Ethiopia

Category	***Richness***	***Shannon***	***Evenness***
Altitudinal gradients			
Low	*7.54[a]±0.59*	*1.00[a]±0.06*	*0.53[a]±0.03*
Middle	*7.42[a]±0.77*	*1.07[a]±0.09*	*0.57[a]±0.04*
High	*7.75[a]±0.76*	*0.97[a]±0.08*	*0.55[a]±0.05*
Mean	7.57±0.40	1.02±0.05	0.55±0.02

Wealth status			
Poor	*4.75^c±0.42*	*0.84^b±0.06*	*0.61^a±0.04*
Medium	*7.58^b±0.46*	*1.07^a±0.09*	*0.53^a±0.04*
Rich	*10.38^a±0.66*	*1.14^a±0.07*	*0.50^a±0.03*
Mean	7.57±0.40	1.02±0.05	0.55±0.02

Key- *Different letters superscripted on the mean indicate significant different (P < 0.05) between the groups, SE- standard error*

3.2. Woody species diversity

The Shannon diversity index and Evenness/Equitablity index were analyzed along an altitudinal gradient and wealth status. Both diversity indices were not significant (P < 0.05) between altitudinal gradients. The highest mean Shannon (1.07±0.09) and Evenness (0.57±0.04) index was observed at middle altitude. The lowest Shannon (0.97±0.08) was recorded at high altitudes whereas the lowest Evenness (0.53±0.03) was recorded at low altitudes. Similar to species richness, the mean Shannon diversity index was increased with household wealth status whereas evenness showed the reverse. Shannon diversity index was significant (P < 0.05) between poor and rich households while evenness didn't show a significant difference between wealth (Table 2). Pearson correlation result showed no relation between species richness and altitude, and a positively significant correlation was seen between species richness and wealth status. There was a significant positive correlation between the Shannon diversity index and wealth status (Table 3).

Table 3: Pearson correlation between altitudinal gradient, wealth status and woody species diversity in homegarden agroforestry systems

Category	*Altitudinal gradients*	*Wealth status*	*Species richness*	*Shannon diversity index*
Wealth status	*0.000*	-	-	-
Species richness	*0.025*	*0.674***	-	-
Shannon diversity index	*-0.028*	*0.319***	*0.470***	-
Evenness	*0.055*	*-0.225*	*-0.270**	*0.610***

*Note: *- significant different (P < 0.05), **- significant different (P < 0.01)*

3.3. Woody species similarity index

The calculated similarity index result indicates that about 51% of the woody species are similar along altitudinal gradients and wealth classes (Fig. 2). The highest similarity index observed between high and middle altitude (55%) and the lowest (45%) registered between high and low altitude. For a wealth class, the highest (60%) similarity was seen between high and medium while the lowest (40%) seen between medium and poor classes (Table 4). On the other hand, 12, seven and three woody species were unique to high, middle and low altitudes respectively whereas 10, six and two species were recorded for rich, medium and poor households respectively (Fig. 2).

Table 4: Woody species similarity index in homegarden agroforestry systems across altitudinal gradient and wealth status

Altitudinal gradient	***High***	***Middle***	***Low***
Middle	*0.55*	-	-
Low	*0.45*	*0.47*	-
Wealth status	***Rich***	***Medium***	***Poor***
Medium	*0.60*	-	-

Poor	*0.44*	*0.40*	-

3.4. Woody species density

Woody species stem density was calculated along altitudinal gradient and wealth status. The mean stem density per homegarden and hactare was (89.06±9.25) and (1236.22±131.42) respectively. Mean stem density was significantly different ($P < 0.001$) between wealth classes. The highest (134.88±18.87) stem density was recorded in the rich followed by medium (87.08±14.35) and poor (45.21±7.38) wealth classes. However, there is no significant difference between stem density and altitudinal gradient at the garden and hectare level (Table 5).

3.5. Basal area distribution

The mean basal area was highest at middle altitude followed by high and low altitude respectively although no significant difference between each altitude. Homegarden level basal area was 1.53±0.29 for the rich, 0.90±0.27 for the medium, and 0.33±0.08 for the poor in which a significant difference ($P < 0.01$) was seen between the poor and rich classes (Table 5). However, there was no significant difference in basal area per hectare (m^2 ha^{-1}) along elevation gradients and wealth status. Finally, the overall basal area distributions of the top ten woody species are presented in Figure 3. *Persea americana* had the highest basal area (886.87 m^2 ha^{-1}) followed by *Cordia africana* (146.48 m^2 ha^{-1}), *Coffea arabica* (130.87 m^2 ha^{-1}), *Mangifera indica* (95.68 m^2 ha^{-1}), *Catha edulis* (64.98 m^2 ha^{-1}), *Grevillea robusta* (46.30 m^2 ha^{-1}), *Citrus sinensis* (18.01 m^2 ha^{-1}), *Psidium guajava* (16.79 m^2 ha^{-1}), *Carica papaya* (15.69 m^2 ha^{-1}) and *Albizia gummifera* (6.51 m^2 ha^{-1}) (Fig. 3).

3.6. Importance value index

The overall importance value index in the entire homegarden agroforestry system value lay between 0.20 to 69.06% for less to more important woody species respectively. However, a large number (29) of woody species had an importance value index of less than 1% (Appendix, 1). Figure 4 indicates the importance value index distribution of the top ten woody species. Accordingly, the importance value of these species was presented as *Catha edulis* (69.06%), *Coffea arabica* (51.01%), *Persea americana* (40.84%), *Cordia africana* (18.07%), *Mangifera indica* (15.81%), *Grevillea robusta (11.98%), Psidium guajava (9.31%), Citrus sinensis (9.17%), Ricinus communis (7.97%)* and *Carica papaya* (6.87%) in their descending order (Fig. 4).

Table 5: Mean (±SE) woody density and basal area distribtution along altitudinal gradients and wealth status in homegarden agroforestry systems

Category	***Density/garden***	***Density/ha***	***BA/garden***	***BA/ha***
Altitudinal gradients				
Low	*82.04^{a}±14.44*	*1151.88^{a}±140.63*	*0.64^{a}±0.13*	*8.97^{a}±1.49*
Middle	*79.88^{a}±12.10*	*1298.50^{a}±275.63*	*1.28^{a}±0.31*	*15.00^{a}±3.49*
High	*105.25^{a}±20.48*	*1258.29^{a} ±252.41*	*0.84^{a}±0.28*	*11.29^{a}±3.79*
Wealth status				
Poor	*45.21^{c}±7.38*	*1654.29^{a}±268.98*	*0.33^{b}±0.08*	*10.59^{a}±2.71*
Medium	*87.08^{b}±14.35*	*1046.13^{ab}±169.67*	*0.90^{ab}±0.27*	*11.37^{a}±1.70*
Rich	*134.88^{a}±18.87*	*1008.25^{b}±217.68*	*1.53^{a}±0.29*	*12.09^{a}±3.65*

Key: Different letters superscripted on the mean indicate significant different ($P < 0.05$) between the groups, SE- standard error

4. DISCUSSION

Woody species composition and diversity significantly vary between wealth statuses, but not between altitudinal gradients. Species richness and Shannon diversity index had shown a significant increasing trend with increasing wealth status. Similarly, richness and Shannon diversity index increase with increasing wealth status in traditional agroforestry practices of south-central Ethiopia (Tadesse *et al*., 2021; Molla *et al*., 2023). Evenness had shown the reverse trend with richness in wealth classes. The result indicates that homegardens with few numbers of different species (lower species richness) have a large abundance for each species (higher evenness). However, there was no continuous increasing or decreasing values of diversity indices for altitudinal gradients. Likewise, species richness and diversity of agroforestry didn't show a parallel relationship with elevation gradient (Tesfay *et al*., 2023). *Catha edulis, Coffea arabica* and *Persea americana* are the most abundant and frequently recorded woody species. The study conducted by Gebrewahid & Abrehe (2019) reported that the distributions of farm woody species are determined by the economic or ecological values they provide. Therefore, the main reason for the higher abundance and distribution of these woody species may be due to the farmer's focus on the production of highly economic and ecologically valued woody species in the present study area.

The number of woody species recorded in the present study area is comparable with woody species (55) reported in traditional agroforestry practices of Dellomenna district, Ethiopia (Molla & Kewessa, 2015). The number is lower than 120 trees and shrubs species reported from southern Ethiopia (Abebe, 2005) and 60 woody species around Jimma Town, southwestern Ethiopia (Wari *et al*., 2019) with similar agroecology. It's also lower than 83 plant species from

eastern Amazonia (Cardozo *et al*., 2015), 138 from south-central Ethiopia (Tadesse *et al*., 2021), 171 from central Nepal (Pandey *et al*., 2021), and 206 from southern and southwestern Ethiopia (Kassa *et al*., 2023). However, it's higher than 53, 36, 49 and 32 woody species reported from Shewarobit district, Ethiopia (Woldeamanual *et al*., 2018), Shashemene district, Ethiopia (Jegora *et al*., 2019), southern Ethiopia (Birhane *et al*., 2020) and southern Tigray, Ethiopia (Eyasu *et al*., 2020) respectively. Also higher than 32 woody species in western Cameroon (Temgoua *et al*., 2020) and 30 woody species in mid-highlands of southern Ethiopia (Tesfay *et al*., 2023) from coffee-based agroforestry systems.

The present study registered 56.4% indigenous and 43.6% exotic whereas 85% indigenous and 15% exotic reported in Dellomenna district, southeastern Ethiopia (Molla & Kewessa, 2015). Comparatively, a high percentage of exotic woody species observed in the study area indicates that the local community gave attention to exotic fruit trees like *Persea americana* and *Mangifera indica* which are accessed in the majority of the sampled homegardens. Similarly, the study conducted by Kassa *et al*. (2023) also reported 69.52% indigenous and 31.48% exotic in homegarden agroforestry systems from similar agroecology in Ethiopia. Similar to this study, *Erythrina brucei* and *Millettia ferruginea* were also reported as the two endemic woody species in Ethiopia in traditional agroforestry practices (Bajigo & Tadesse, 2015).

The variation in the woody species similarity index recorded was not only due to elevation change but also wealth status which may be affected by the farmer's homegarden size, extension service, improved seedlings, and labor forces. Likewise, the species similarity index between the sites changed with elevation and woody production (Chemeda *et al*., 2022). The highest similarity index reported between high and middle altitudes agreed with Tesfay *et al* (2023), who

reported the highest species similarity between adjacent elevations in coffee-based agroforestry in Ethiopia. Temgoua *et al*. (2020) reported the highest woody species similarity due to closed ecological conditions between coffee agroforestry systems and forests in Cameroon.

The mean stem density was significantly increased with wealth status with no significant difference between altitudinal gradients. This indicates that household wealth status determines woody species density in homegarden agroforestry systems. The present study reported the top ten woody species having the highest basal area. Of which, *Persea americana* an exotic fruit tree species has the highest (61.52%) basal area. However, Molla *et al*. (2023) reported that *Eucalyptus camaldulensis* had the highest (30.03%) basal area among the top ten woody species basal area registered in traditional agroforestry practices of Wondo district, south-central Ethiopia. The highest importance value index was recorded for *Catha edulis* followed by *Coffea arabica* and *Persea americana.* Similarly, *Catha edulis* was reported as an economically important cash crop among woody species of homegardens in Ethiopia (Gebrehiwot *et al*., 2016). On the contrary, *Eucalyptus camaldulensis* (Abebe 2005; Molla *et al*., 2023), *Faidherbia albida* (Guyassa & Raj, 2013), and *Cordia africana* (Legesse & Negash, 2021) were reported as the most important woody species in homegarden agroforestry systems in Ethiopia.

5. CONCLUSION

Homegarden agroforestry systems of southwestern Ethiopia are rich in woody species diversity. Households within a rich wealth class had the highest number of woody species compared to medium and poor wealth classes. Woody species richness was not statistically significant between altitudinal gradients. Shannon diversity index significantly increases with increasing wealth status with no significant difference between altitudinal gradients. The finding indicates

that household economic status determines the diversification of woody species in ecologically related human-managed land use sytems. Therefore, concerned bodies should be provided the necessary materials that support local communities in homegarden agroforestry systems woody species diversification. Additionally, further investigation could be needed to know details of factors that affect woody species composition and diversity in homegarden agroforestry and its sustainable socio-economic and ecological uses.

REFERENCES

Abebe, T. (2005). Diversity in homegarden agroforestry system of southern Ethiopia. PhD Dissertation, Wageningen Agricultural University. The Netherlands.

Abebe, T., Wiersum, K.F & Bongers, F. (2010). Spatial and temporal variation in crop diversity in agroforestry homegardens of southern Ethiopia. *Agroforest Syst*, **78**:309–322.

Abebe, T., Wiersum, K.F., Bongers, F & Sterck, F. (2006). Diversity and dynamics in homegardens of southern Ethiopia. Kumar, B.M & Nair, P.K.R, (eds). Tropical homegardens: a time-tested example of sustainable agroforestry, Dordrecht: Springer Science. pp. 123–142.

Asfaw, B & Lemenih, M. (2010). Traditional agroforestry systems as a safe haven for woody plant species: a case study from a topo-climatic gradient in South Central Ethiopia. *Forests, Trees and Livelihoods*, ***19***(4): 359-377.

Azene B., Birnie, A & Tengnäs, B. (1993). Useful trees and shrubs for Ethiopia: Identification, propagation and management for agricultural and pastoral communities. SIDA's regional Soil Conservation Unit. Ethiopia. P. 474.

Bajigo, A & Tadesse, M. (2015). Woody species diversity of traditional agroforestry practices in Gununo watershed in Wolayitta zone, Ethiopia. *Forest Research*, **4**(4): 2168-9776.

Bardhan, S., Jose, S., Biswas, S., Kabir, K & Rogers, W. (2012). Homegarden agroforestry systems: an intermediary for biodiversity conservation in Bangladesh. *Agroforest Syst*, **85**:29–34.

Bayala, J., Kindt, R., Belem, M & Kalinganire, A. (2011). Factors affecting the dynamics of tree diversity in agroforestry parklands of cereal and cotton farming systems in Burkina Faso. *New Forests*, **41**:281–296.

Beetz, A.E. (2011). Agroforestry: An Overview. The National Sustainable Agriculture Information Service, ATTRA, http://www.attra.ncat.org

Bhagwat, S., Willis, K., Birks, H & Whittaker, R. (2008). Agroforestry: a refuge for tropical biodiversity? *Trends Ecology and Evol*ution, **23**: 261–267.

Birhane, E., Ahmed, S., Hailemariam, M., Negash, M., Rannestad, M.M & Norgrove, L. (2020). Carbon stock and woody species diversity in homegarden agroforestry along an elevation gradient in southern Ethiopia. *Agroforest Syst*, https://doi.org/10.1007/s10457-019-00475-4.

Bishaw, B & Abdelkadir, A. (2003). Agroforestry and Community Forestry for Rehabilitation of Degraded Watersheds on the Ethiopian Highlands. International Conference on African Development Archives. 78. https://scholarworks.wmich.edu/africancentericadarchive/78.

Bucheli, V.J.P & Bokelmann, W. (2017). Agroforestry systems for biodiversity and ecosystem services: the case of the Sibundoy Valley in the Colombian province of Putumayo. *International Journal of Biodiversity Science*, *Ecosystem Services & Management*, **13**(1): 380-397.

Cardozo, E.G., Muchavisoy, H.M., Silva, H.R., Zelarayán, M.L.C., Leite, M.F.A., Rousseau, G.X & Gehring, C. (2015). Species richness increases income in agroforestry systems of eastern Amazonia. *Agroforestry systems*, **89**: 901-916.

Chemeda, B.A., Wakjira, F.S & Hizikias, E.B. (2022). Tree diversity and biomass carbon stock analysis along altitudinal gradients in coffee based agroforestry system of Western Ethiopia. *Cogent Food & Agriculture*, **8**(1): 2123767.

Dawson, I.K., Guariguata, M.R., Loo, J., Weber, J.C., Lengkeek, A., Bush, D., Cornelius, J., Guarino, L., Kindt, R., Orwa, C., Russell, J & Jamnadass, R. (2013). What is the relevance of smallholders'

agroforestry systems for conserving tropical tree species and genetic diversity in circa situm, in situ and ex situ settings? A review. *Biodivers Conserv*, **22**(2): 301–324.

Edwards, S., Tadesse, M & Hedberg, I. (1995). Flora of Ethiopia and Eritrea, Vol. 2, Part 2, The National Herbarium, Addis Ababa University, Addis Ababa, Ethiopia and Department of Systematic Botany, Uppsala University, Uppsala, Sweden.

Endale, Y., Derero, A., Argaw, M & Muthuri, C. (2017). Farmland tree species diversity and spatial distribution pattern in semi-arid East Shewa, Ethiopia. *Forests, Trees and Livelihoods*, **26**(3): 199-214. https://doi.org/10.1080/14728028.2016.1266971

Gebremedhin, T., & Mulubrehan, H. (2007). Women in backyards: root crop production and biodiversity management in backyards. A case study in five selected woredas of tigray regional state, Northern Ethiopia. Northern Ethiopia DCG Report No, 50.

George, M. V & Christopher, G. (2020). Structure, diversity and utilization of plant species in tribal homegardens of Kerala, India. *Agroforestry systems*, ***94***(1): 297-307.

Sood, K.K & Mitchell, C.P. (2009). Identifying important biophysical and social determinants of on-farm tree growing in subsistence-based traditional agroforestry systems. *Agroforest Syst*, **75**:175–187.

Guyassa, E & Raj, A.J. (2013). Assessment of biodiversity in cropland agroforestry and its role in livelihood development in dryland areas: A case study from Tigray region, Ethiopia. *International Journal of Agricultural Technology,* **9**(4): 829-844.

Harvey, C.A & Villalobos, J.A.G. (2007). Agroforestry systems conserve species-rich but modified assemblages of tropical birds and bats. *Biodiversity and Conservation*, **16**: 2257-2292.

Hedberg, I & Edwards, S. (1995). Flora of Ethiopia and Eritrea Vol. 7. Poaceae. The National Herbarium, Addis Ababa, Ethiopia, and Department of Systematic Botany, Uppsala, Sweden.

Hedberg, I., Edwards, S & Nemomissa, S. (2003). Flora of Ethiopia and Eritrea Vol. 4, Part 1. Apiaceae to Dipsacaceae. The National Herbarium, Addis Ababa, Ethiopia, and Department of Systematic Botany, Uppsala, Sweden.

Hedberg, I., Ensermu Kelbessa, Edwards, S., Sebsebe Demissew & Persson, E. (2006). Flora of Ethiopia and Eritrea Vol. 5. Gentianaceae to Cyclocheilaceae. The National Herbarium, Addis Ababa University, Addis Ababa, Ethiopia and Department of Systematic Botany, Uppsala University, Uppsala, Sweden.

Henry, M., Tittonell, P., Manlay, R.J., Bernoux, M., Albrecht, A & Vanlauwe, B. (2009). Biodiversity, carbon stocks and sequestration potential in aboveground biomass in smallholder farming systems of western Kenya. *Agriculture, ecosystems & environment*, ***129***(1-3): 238-252.

Jama, B., Elias, E & Mogotsi, K. (2006). Role of agroforestry in improving food security and natural resource management in the drylands: a regional overview. *Journal of the Drylands,* **1**(2): 206-211.

Jose, S. (2009). Agroforestry for ecosystem services and environmental benefits: an overview. *Agroforestry Systems*, **76**:1–10.

Kassa, G., Bekele, T., Demissew, S & Abebe, T. (2023). Plant species diversity, plant use, and classification of agroforestry homegardens in southern and southwestern Ethiopia. *Heliyon*, **9**.

Kent, M & Coker, P. (1992). Vegetation description and analysis. A practical approach. London: Belhaven Press.

Korwar, G.R., Prasad, J.V.N.S., Rajeshwara Rao, G., Venkatesh, G., Pratibha, G & Venkateswarlu, B. (2018). Agroforestry as a Strategy for Livelihood Security in the Rainfed Areas: Experience and Expectations. In J.C. Dagar *et al*. (eds.). Agroforestry Systems in India: Livelihood Security & Ecosystem Services. *Advances in Agroforestry*, **10**. https://10.1007/978-81-322-1662-9_5.

Krebs, C.J. (1989). Ecological methodology. New York: Harper Collins Publishers.

Lamprecht, H. (1989). Silviculture in the tropics. Tropical forest ecosystems and their tree species possibilities and methods for their long term utilization. T2-verlagsgeslls chaft, RoBdort, Germany.

Leakey, R.R.B. (2017). Definition of agroforestry revisited. In: Multifunctional Agriculture – Achieving Sustainable Development in Africa, RRB Leakey, 5-6, Academic Press, San Diego, California, USA.

Legesse, A & Negash, M. (2021). Species diversity, composition, structure and management in agroforestry systems: the case of Kachabira district, Southern Ethiopia. *Heliyon*, **7**(3): e06477.

Magurran, A.E. (2004). Measuring biological diversity. Malden and Oxford: Blackwell Publishing.

Maroyi, A. (2009). Traditional homegardens and rural livelihoods in Nhema, Zimbabwe: a sustainable agroforestry system. *International Journal of Sustainable Development & World Ecology*, **16**(1): 1-8.

Mekonnen, E.L., Asfaw, Z & Zewudie, S. (2014). Plant species diversity of homegarden agroforestry in Jabithenan District, North-Western Ethiopia. *Int. J. Biodivers. Conserv.* ***6***(4): 301-307.

Mohan, S. (2004). An assessment of the ecological and socioeconomic benefits provided by homegardens: a case study of Kerala, India. University of Florida.

Molla, T., Asfaw, A., Muluneh, M.G & Worku, B.B. (2023). Diversity of woody species in traditional agroforestry practices in Wondo district, south-central Ethiopia. *Heliyon*, **9** e13549. https://doi.org/10.1016/j.heliyon.2023.e13549

Montagnini, F. (2020). The Contribution of Agroforestry to Restoration and Conservation: Biodiversity Islands in Degraded Landscapes. In: J. C. Dagar *et al*. (eds.), Agroforestry for Degraded Landscapes: Recent Advances and Emerging Challenges - Vol. 1, https://doi.org/10.1007/978-981-15-4136-0_15

Montagnini, F., Francesconi, W & Rossi, E. (ed.) (2011). Agroforestry as a tool for landscape restoration. Nova Science, New York.

Motuma, T., Zebene, A., Mulugeta, L & Karltun, E. (2008). Woody species diversity in a changing landscape in the south-central highlands of Ethiopia. *Agriculture, Ecosystems and Environment*, **128**: 52–58.

Nair, P.K.R. (1993). An Introduction to Agroforestry. Kluwer Academic Publishers-ICRAF. P. 489.

Nair, P.K.R. (2017). Managed Multi-strata Tree + Crop Systems: An Agroecological Marvel. *Front. Environ. Sci*. **5**:88. doi: 10.3389/fenvs.2017.00088.

Pandey, H.P., Pokhrel, N.P., Luitel, D.R., Acharya, K & Shah, K.K. (2021). Diversity of agroforestry species and uses in two ecological regions: A Case from Central Nepal. *Advances in Agriculture*, ***2021***: 1-9.

R Core Team. (2021). R: A language and environment for statistical computing. R Foundation for Statistical Computing, Vienna, Austria. URL, https://www.R-project.org/.

Reang, D., Hazarika, A., Sileshi, G.W., Pandey, R., Das, A.K & Nath, A.J. (2021). Assessing tree diversity and carbon storage during land use transitioning from shifting cultivation to indigenous agroforestry systems: Implications for REDD+ initiatives. *Journal of Environmental Management*, **298**: 113470.

Rendón-Sandoval, F.J., Casas, A., Moreno-Calles, A.I., Torres-García, I & García-Frapolli, E. (2020). Traditional Agroforestry Systems and Conservation of Native Plant Diversity of Seasonally Dry Tropical Forests. *Sustainability*, **12**: 4600; doi:10.3390/su12114600.

Schroth G., Fonseca, G.A.B., Harvey, C.A., Gascon, C., Vasconcelos, H.L & Izac, A.M.N. (2004). Agroforestry and Biodiversity Conservation in Tropical Landscapes. Island Press, Washington.

Tadesse, E., Abdulkedir, A., Khamzina, A., Son, Y & Noulèkoun, F. (2019). Contrasting Species Diversity and Values in Home Gardens and Traditional Parkland Agroforestry Systems in Ethiopian Sub-Humid Lowlands. *Forests*, **10**(266).

Tadesse, E., Negash, M & Asfaw, Z. (2021). Impacts of traditional agroforestry practices, altitudinal gradients and households' wealth status on perennial plants species composition, diversity, and structure in south-central Ethiopia. *Agroforestry Systems*, ***95***: 1533-1561.

Temgoua, L.F., Etchike, A.B.D., Solefack, M.C.M., Tumenta, P & Nkwelle, J. (2020). Woody species diversity conservation and carbon sequestration potential of coffee agroforestry systems in the Western Region of Cameroon. *Journal of Horticulture and Forestry*, **12**(2): 35-48.

Tesfay, F., Moges, Y & Asfaw, Z. (2022). Woody species composition, structure, and carbon stock of coffee-based agroforestry system along an elevation gradient in the moist mid-highlands of Southern Ethiopia. *International Journal of Forestry Research, 2022.*

Torquebiau, E & Penot, E. (2006). Ecology versus economics in tropical multistrata agroforests. In: B.M. Kumar & P.K.R. Nair (eds). Tropical homegardens: A time-tested example of sustainable agroforestry, 269-282.

van Noordwijk, M., Rahayu, S., Gebrekirstos, A., Kindt, R., Tata, H.L., Muchugi, A., Ordonnez, J.C & Xu, J. (2019). Tree diversity as basis of agroforestry. In: van Noordwijk M, ed. Sustainable development through trees on farms: agroforestry in its fifth decade. Bogor, Indonesia: World Agroforestry (ICRAF) Southeast Asia Regional Program. pp 17−44.

Wari, B.N., Feyssa, D.H & Kebebew, Z. (2019). Assessment of woody species in agroforestry systems around Jimma Town, Southwestern Ethiopia. *International Journal of Biodiversity and Conservation*, **11**(1): 18-30.

Woldeamanual, W., Asfaw, Z & Lemessa, D. (2018). The Efect of the Surrounding Landscape and Socioeconomic characteristics on Woody Species Diversity in Homegardens of Shewarobit District, Northeast Ethiopia. *Small-scale Forestry*, https://doi.org/10.1007/s11842-018-9398-1

Appendix 1: List of woody species scientific, local and family name, and their origin in homegarden agroforestry systems of southwest Ethiopia

**№**	***Scientific name***	***Local name***	***Family name***	***Origin***
1	*Acacia abyssinica Hochst. ex Benth.*	*Sondii*	*Fabaceae*	*I*
2	*Albizia gummifera (J. F. Gmel.) C.A. Sm.*	*Ambabbeessa*	*Fabaceae*	*I*
3	*Albizia schimperiana Oliv.*	*Ambabbeessa arba*	*Fabaceae*	*I*

4	*Allophylus abyssinicus (Hochst.) Radikofer*	*Sehoo*	*Sapindaceae*	*I*
5	*Annona senegalensis pers.*	*Gishxaa*	*Annonaceae*	*I*
6	*Artocarpus heterophyllus*	*Jakifiruttii*	*Moraceae*	*E*
7	*Azadirachta indica A.Juss.*	*Niimii*	*Meliaceae*	*E*
8	*Borassus aethiopum Mart.*	*Meexxii*	*Arecaceae*	*I*
9	*Brucea antidysenterica J.F.Miller*	*Qomonyoo*	*Simaroubaceae*	*I*
10	*Calpurnia aurea (Ait.) Benth.*	*Ceekaa*	*Fabaceae*	*E*
11	*Carica papaya L.*	*Papaayee*	*Caricaceae*	*I*
12	*Casimiroa edulis LaLava*	*Kashimiirii*	*Rutaceae*	*E*
13	*Casuarina equisetifolia*	*Shuwashuwee*	*Casuarinaceae*	*E*
14	*Catha edulis (Vahl) Forssk. ex Endl.*	*Caatii*	*Celastraceae*	*I*
15	*Citrus aurantifolia (Christm.) Swingle*	*Loomii*	*Rutaceae*	*E*
16	*Citrus medica*	*Maraaraa*	*Rutaceae*	*E*
17	*Citrus sinensis*	*Burtukaana*	*Rutaceae*	*E*
28	*Coffea arabica L*	*Buna*	*Rubiaceae*	*I*
19	*Cordia africana Lam.*	*Waddeessa*	*Boraginaceae*	*I*
20	*Croton macrostachyus Hochst ex Del.*	*Makkannisa*	*Euphorbiaceae*	*I*
21	*Cupressus lusitanica*	*Gattiraa faranjii*	*Cupressaceae*	*E*
22	*Dovyalis abyssinica (A. Rich.) Warb.*	*Koshimii*	*Flacourtiaceae*	*I*
23	*Dracaena fragrans*	*Omoo*	*Dracaenaceae*	*I*
24	*Dracaena steudneri Engl.*	*Bubbiftuu*	*Dracaenaceae*	*I*
25	*Duranta erecta L.*	*Ababaa*	*Verbenaceae*	*E*
26	*Ehretia cymosa Thonn.*	*Ulaagaa*	*Boraginaceae*	*I*
27	*Erythrina abyssinica Lam. ex DC.*	*Waleensuu*	*Fabaceae*	*I*
28	*Erythrina brucei Schweinf.*	*Beroo*	*Fabaceae*	*I*®
29	*Eucalyptus camaldulensis*	*Baargamo diimaa*	*Myrtaceae*	*E*
30	*Eucalyptus citriodora Hook.*	*Baargamo*	*Myrtaceae*	*E*

31	*Eucalyptus globules Labill.*	*Baargamo adii*	*Myrtaceae*	*E*
32	*Ficus thonningii Blume*	*Dambii*	*Moraceae*	*I*
33	*Ficus sur Forssk.*	*Harbuu*	*Moraceae*	*I*
34	*Grevillea robusta A.Cunn. ex R.Br.*	*Giraavillaa*	*Proteaceae*	*E*
35	*Jacaranda mimosifolia D.Don*	*Jakarandaa*	*Bignoniaceae*	*E*
36	*Maesa lanceolata Forssk .*	*Abbayyii*	*Myrsinaceae*	*I*
37	*Malus domestica*	*Appilii*	*Rosaceae*	*E*
38	*Mangifera indica L.*	*Maangoo*	*Anacardiaceae*	*E*
39	*Millettia ferruginea (Hochst.) Baker*	*Askiraa*	*Fabaceae*	*I*®
40	*Moringa oleifera*	*Mooriingaa*	*Moringaceae*	*E*
41	*Morus alba L.*	*Goraa*	*Moraceae*	*E*
42	*Ocimum lamiifolium Hochst. ex Benth*	*Damakasee*	*Lamiaceae*	*I*
43	*Olea europaea (Wall. ex DC.) Cifferri*	*Ejersa*	*Oleaceae*	*I*
44	*Olea welwitschii (Knobl.) Gilg & Schellenb.*	*Bayaa*	*Oleaceae*	*I*
45	*Persea americana Mill.*	*Avokaadoo*	*Lauraceae*	*E*
46	*Pouteria campechiana*	*Eggifiruttii*	*Sapotaceae*	*E*
47	*Prunus persica (L.) Batsch*	*Kuukkoo*	*Rosaceae*	*E*
48	*Psidium guajava L.*	*Zetunaa*	*Myrtaceae*	*E*
49	*Rhamnus prinoides L'Herit*	*Geeshoo*	*Rhamnaceae*	*I*
50	*Ricinus communis L,*	*Qobboo*	*Euphorbiaceae*	*I*
51	*Sapium ellipticum (Hochst) Pax.*	*Bosoqa*	*Euphorbiaceae*	*I*
52	*Sesbania sesban*	*Sasbaniyaa*	*Fabaceae*	*I*
53	*Spathodea campanulata*	*Ispaatoodaa*	*Bignoniaceae*	*E*
54	*Vernonia amygdalina Del.*	*Eebicha*	*Asteraceae*	*I*
55	*Vernonia auriculifera Heirn.*	*Reejjii*	*Asteraceae*	*I*

***Key**: Origin: I- Indigenous, E- Exotic, ®- Endemic species*

Appendix 2: Percentage frequency (%Freq), relative dominance (Rdo), relative density (Rde), relative frequency (RF) and importance value index (IVI) of woody species across the entire homegarden agroforestry systetms

N/S	***Scientific Name***	***% Freq***	***Rdo***	***Rde***	***RF***	***IVI***
1	*Catha edulis*	*54.2*	*7.4*	*54.5*	*7.1*	*69.1*
2	*Coffea arabica*	*84.7*	*10.6*	*29.3*	*11.1*	*51.0*
3	*Persea americana*	*79.2*	*27.5*	*3.0*	*10.4*	*40.8*
4	*Cordia africana*	*44.4*	*11.2*	*1.1*	*5.8*	*18.1*
5	*Mangifera indica*	*44.4*	*9.0*	*1.0*	*5.8*	*15.8*
6	*Grevillea robusta*	*36.1*	*6.3*	*1.0*	*4.7*	*12.0*
7	*Psidium guajava*	*37.5*	*3.8*	*0.6*	*4.9*	*9.3*
8	*Citrus sinensis*	*34.7*	*3.9*	*0.7*	*4.6*	*9.2*
9	*Ricinus communis*	*40.3*	*1.2*	*1.5*	*5.3*	*8.0*
10	*Carica papaya*	*20.8*	*3.7*	*0.5*	*2.7*	*6.9*
11	*Albizia gummifera*	*27.8*	*2.4*	*0.4*	*3.6*	*6.4*
12	*Vernonia auriculifera*	*30.6*	*0.0*	*1.0*	*4.0*	*5.0*
13	*Cupressus lusitanica*	*18.1*	*1.2*	*0.7*	*2.4*	*4.3*
14	*Annona senegalensis*	*18.1*	*1.3*	*0.5*	*2.4*	*4.1*
15	*Duranta erecta*	*23.6*	*0.0*	*0.7*	*3.1*	*3.8*
16	*Croton macrostachyus*	*13.9*	*0.8*	*0.3*	*1.8*	*2.9*
17	*Erythrina brucei*	*11.1*	*0.8*	*0.3*	*1.5*	*2.6*
28	*Vernonia amygdalina*	*12.5*	*0.7*	*0.1*	*1.6*	*2.4*
19	*Jacaranda mimosifolia*	*6.9*	*1.3*	*0.1*	*0.9*	*2.3*
20	*Casimiroa edulis*	*11.1*	*0.7*	*0.2*	*1.5*	*2.4*
21	*Sesbania sesban*	*12.5*	*0.1*	*0.4*	*1.6*	*2.2*
22	*Albizia schimperiana*	*6.9*	*0.8*	*0.1*	*0.9*	*1.8*

23	*Millettia ferruginea*	*4.2*	*1.1*	*0.1*	*0.6*	*1.7*
24	*Calpurnia aurea*	*9.7*	*0.1*	*0.4*	*1.3*	*1.7*
25	*Rhamnus prinoides*	*6.9*	*0.0*	*0.2*	*0.9*	*1.1*
26	*Ehretia cymosa*	*4.2*	*0.4*	*0.1*	*0.6*	*1.0*
27	*Acacia abyssinica*	*1.4*	*0.7*	*0.1*	*0.2*	*0.9*
28	*Malus domestica*	*5.6*	*0.0*	*0.3*	*0.7*	*1.0*
29	*Erythrina abyssinica*	*5.6*	*0.1*	*0.1*	*0.7*	*0.9*
30	*Citrus aurantifolia*	*5.6*	*0.1*	*0.1*	*0.7*	*0.9*
31	*Olea europaea*	*4.2*	*0.2*	*0.1*	*0.6*	*0.8*
32	*Casuarina equisetifolia*	*2.8*	*0.4*	*0.0*	*0.4*	*0.8*
33	*Eucalyptus citriodora*	*2.8*	*0.2*	*0.1*	*0.4*	*0.7*
34	*Eucalyptus globulus*	*2.8*	*0.2*	*0.1*	*0.4*	*0.6*
35	*Dovyalis abyssinica*	*2.8*	*0.1*	*0.1*	*0.4*	*0.5*
36	*Azadirachta indica*	*2.8*	*0.1*	*0.0*	*0.4*	*0.5*
37	*Olea welwitschii*	*2.8*	*0.1*	*0.0*	*0.4*	*0.5*
38	*Citrus medica*	*2.8*	*0.1*	*0.0*	*0.4*	*0.5*
39	*Dracaena steudneri*	*1.4*	*0.3*	*0.0*	*0.2*	*0.5*
40	*Artocarpus heterophyllus*	*6.9*	*0.2*	*0.0*	*0.2*	*0.5*
41	*Maesa lanceolata*	*2.8*	*0.1*	*0.0*	*0.4*	*0.5*
42	*Moringa oleifera*	*1.4*	*0.2*	*0.0*	*0.2*	*0.4*
43	*Morus alba*	*2.8*	*0.0*	*0.0*	*0.4*	*0.4*
44	*Borassus aethiopum*	*1.4*	*0.2*	*0.0*	*0.2*	*0.4*
45	*Allophylus abyssinicus*	*1.4*	*0.2*	*0.0*	*0.2*	*0.4*
46	*Pouteria campechiana*	*1.4*	*0.1*	*0.0*	*0.2*	*0.3*
47	*Ficus sur*	*1.4*	*0.1*	*0.0*	*0.2*	*0.3*
48	*Dracaena fragrans*	*1.4*	*0.1*	*0.1*	*0.2*	*0.3*
49	*Ficus thonningii*	*1.4*	*0.1*	*0.0*	*0.2*	*0.3*

50	*Eucalyptus camaldulensis*	*1.4*	*0.1*	*0.0*	*0.2*	*0.3*
51	*Prunus persica*	*1.4*	*0.1*	*0.0*	*0.2*	*0.3*
52	*Spathodea campanulata*	*1.4*	*0.1*	*0.0*	*0.2*	*0.3*
53	*Sapium ellipticum*	*1.4*	*0.1*	*0.0*	*0.2*	*0.3*
54	*Ocimum lamiifolium*	*1.4*	*0.0*	*0.0*	*0.2*	*0.2*
55	*Brucea antidysenterica*	*1.4*	*0.0*	*0.0*	*0.2*	*0.2*

List of figures

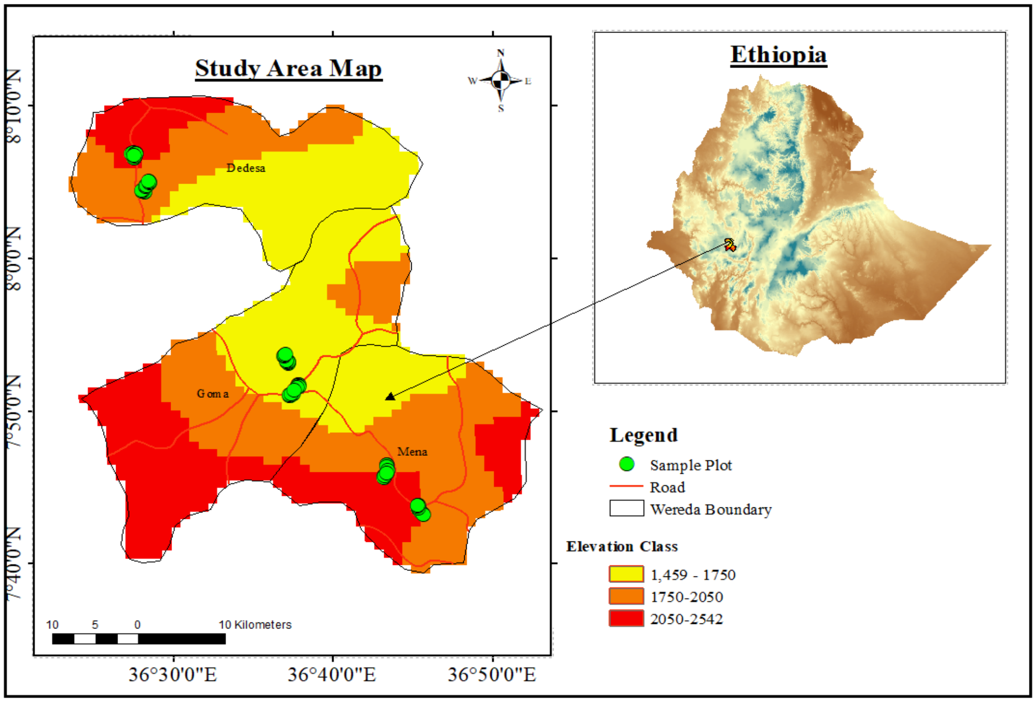

Figure 1: Map of the Study Area

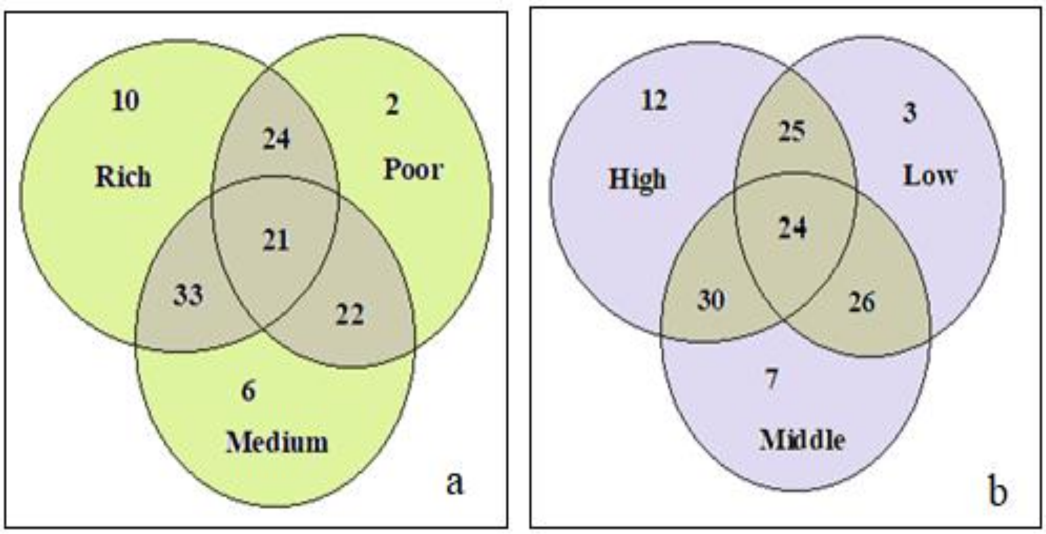

Figure 2: Venn diagram representation of the similarity and dissimilarity of woody species across (a) wealth status and (b) altitudinal gradient in homegarden agroforestry systems

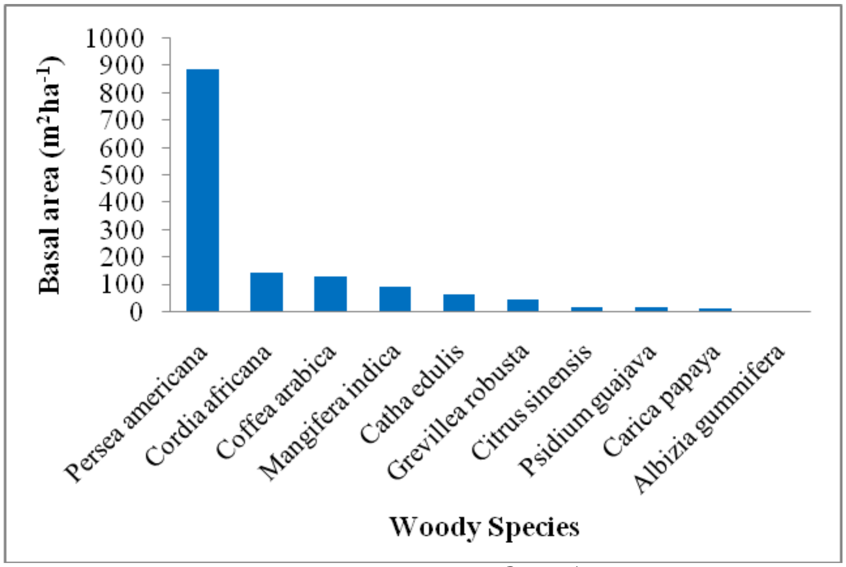

Figure 3: Basal area distribution (m^2 ha^{-1}) for the top ten woody species in homegarden agroforestry systems at southwest Ethiopia

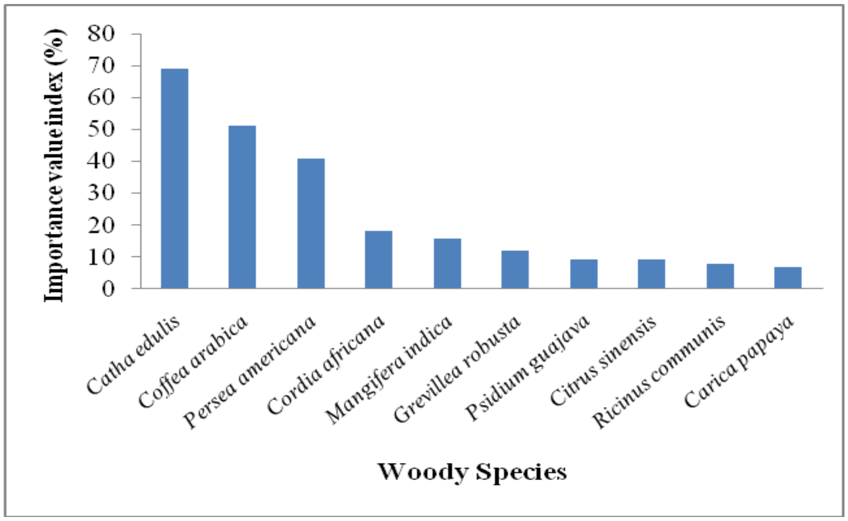

Figure 4: Importance value index of the top ten woody species across the entire homegarden agroforestry systems

Woody species composition and diversity of ~~homegarden agroforestry~~agroforestry homegardens along altitudinal gradient in southwest Ethiopia

Tefera Jegora[1, 2,*], Kitessa Hundera[1], Zerihun Kebebew[1]and Adugna Eneyew[1]

[1]College of Agriculture and Veterinary Medicine, Jimma University, Ethiopia; [2]College of Agriculture and Forestry, Mettu University, Ethiopia, *- Corresponding author e-mail: teferajegora@gmail.com (TJ)

Abstract

Homegarden agroforestry systems that integrate trees with agricultural practices are usually ~~known~~ valued for the conservation of farm biodiversity. Despite the system having a significant conservation role, ~~limited information was revealed~~litle is known on woody species composition and diversity following the elevation belt of southwest Ethiopia. A complete enumeration of 72 homegardens (24 each from altitudinal gradient) was purposively selected for woody species inventory. A total of 55 woody species belonging to 31 families and 45 genera were recorded. Of which, 56.4% of woody species are indigenous and two are endemic to Ethiopia. Family Fabaceae was the most represented family with eight species~~Fabaceae is the most dominant woody having eight species~~. The highest species richness (42) was observed at high altitudes followed by 39 species at middle and 31 species at low altitudes but no significant difference between them. Species richness significantly ($P < 0.001$) increased with increasing wealth status. The overall richness distribution was 46, 40, and 27 across rich, medium, and poor wealth classes respectively. Shannon diversity index differed significantly~~was significant~~ between poor and rich households. Pearson correlation result shows a strong positive and significant

correlation between richness and wealth status. The mean woody species density was 89.06±9.25 and 1236.22±131.42 per garden and hectare, respectively. Stem density was significantly higher~~showed a significant difference~~ (P < 0.001) ~~between wealth statuses~~in wealthier farms. ~~Therefore, the household economy significantly determines woody species composition and diversity of homegarden agroforestry systems in southwest Ethiopia~~We found that, in southwestern Ethiopia wealthier agroforestry farms can support more woody species diversity in homegardens.

Key words: Altitudinal change, species richness, wealth status, woody diversity

Background

Agroforestry ~~system~~ is a strategic land use plan to conserve biodiversity in deforested and fragmented landscapes [1-3]. The practice integrates the most environmentally sound forestry and agricultural systems into the same land management unit with sustainable and profitable interaction between trees, crops, and livestock~~trees-crops-livestock~~ [4]. It supports the floral and faunal assemblages that can be as species-rich, abundant, and diverse as forests with modified and non-forest species compositions [5, 6]. These help agroforestry systems to have diverse components and multi-strata structures with multiple ecological and socio-economic benefits [7-9].

Agroforestry is a potential biodiversity conservation site following ~~the~~ intact natural forests as it provides an important reservoir~~s~~ for terrestrial biodiversity [10, 11]. Tropical agroforestry systems have high levels of woody species diversity that are naturally retained and/or planted on farmland [12-14]. A diversified woody species in the homegarden agroforestry systems of

southwest Ethiopia is one of these dominant practices, which is dominated by khat and coffee shrubs. *Coffea arabica* and *Catha edulis* are the two most important income-generting woody species of homegardes in southern Ethiopia [15]). ~~The system~~It encourages tree plantation and maintenance to diversify multipurpose woody species ~~as a means of~~ that support smallholder farmers' livelihoods [16-18]. Agroforestry systems serve as in situ conservations containing high species richness and diversity valued and conserved by the farmers [10, 19]. As a result, agroforestry plays five vital roles in biodiversity conservation: (1) provides habitat for species to tolerate a certain level of disturbance; (2) reduces the rate of conversion of natural habitat by providing more productive and sustainable alternative agricultural systems; (3) provides corridors between habitat remnants and the conservation of area-sensitive floral and faunal species; (4) preserves the germplasm of sensitive species; and (5) provides ecosystem services such as erosion control, increased infiltration capacity, reduced degradation, and habitat fragmentation [3, 20-22].

Homegarden agroforestry is usually managed by ~~the~~ members of the households for the production of subsistence crops and sometimes cash objectives [23]. The diverse and multi-strata components of the system not only provide significant food and income sources [24-27] but also serve as biodiversity conservation [28, 29]. ~~Constantly, it~~It varies from the nearby agroforestry practices due to their mosaic patches or plots dominated by a diversity of woody species [15]. Consisting of ~~Having~~ multiple strata, complex structure, high species richness, evenness, and abundance make homegardens typically similar to natural forests [6, 30]. Homegarden contains high species diversity and usually ~~3-4~~ 3–4 vertical canopy strata, including trees as an upper story, herbaceous vegetation as near ground, and intermediate layers in between [4, 7, 15]. This

composite nature of the agroforestry system is under the influence of different ecological and socio-economic factors globally [14, 31, 32]. For example, [33] revealed that household level of resource endowment significantly affects tree number, species richness, and species diversity.

In Ethiopia, agroforestry is an old farming system containing low to high woody species diversity distribution due to variations in ecological and socio-economic factors such as elevation, size of farmland, and wealth status [15, 25, 34-36]. The results of the present study did not show a significant difference among altitudinal gradients and woody species distribution. However, household wealth variation significantly influences the distribution of woody species composition and diversity in homegarden agroforestry systems. ~~According to [37], woody~~ Woody species composition in the agroforestry system is influenced by household wealth status and other socio-economic attributes such as size of farmland, level of education, family size, and off-farm economic sources [37]. In indigenous agricultural practices of the northern Ethiopian homegardens, [38] reported variations between woody species composition, density, and diversity along altitudinal gradients. Additionally, ~~there is a recommendation by~~ [39] ~~for~~ recommends further investigation into the interaction between homegarden agroforestry woody species and their physical environment in southwestern Ethiopia. Therefore, this study was conducted to ~~deal with the~~ reveal woody species composition and diversity of homegarden agroforestry systems along an altitudinal gradient and household wealth status in southwest Ethiopia.

Materials and methods

Description of the study area

The study was conducted in three districts, namely Mana, Gomma, and Dhidhessa, following the elevation belt of southwest Ethiopia. The area is located between 7°40ˈN-8°10ˈN and 36°30ˈE-36°50ˈE with an elevation range of 1459m to 2542m above sea level (Fig 1). The area received a mean annual rainfall of 1570mm with ~~13-28°C~~ minimum and maximum temperature of 13–28 °C, respectively. It is occupied by cultivated land, wetlands, settlements, commercial farms, shrubland, grassland, and forest land. Agro-ecologically, it is classified into lowland, midland, and highland, with midland-dominated areas. Agriculture is the main source of socio-economic development~~ having~~, with coffee and khat as dominant cash crops. Homegarden agroforestry (coffee, enset and khat)-based systems are the most accredited farming systems.

~~Figure 1:~~Fig 1. Map of the study area. Map of the study area has been created using shapefiles acquired for free from the Ethiopian Mapping Agency. The Digital Elevation Model (SRTM, 30m resolution) can be accessed for free at EarthExplorer (usgs.gov)

Sampling strategy

Woody species inventory was conducted in homegarden agroforestry systems across the elevation belt of southwest Ethiopia. Elevation classification was made purposively based on the presence of homegarden agroforestry systems. Elevation data was gathered from the Digital Elevation Model in Google Earth [40]. Then, three altitudinal gradients were made as low ~~(1459-1750m)~~ (1459–1750 m), middle (1750–2050 m), and high (2050–2542 m) ~~middle (1750-2050m)~~

~~and high (2050-2542m)~~ following the distributions of homegarden agroforestry with its perspective altitude to see the effect of altitudinal change on woody species diversity. Additionally, wealth status was used as it positively or negatively affects woody species in homegarden agroforestry systems and is classified as rich, medium and poor [14, 41]. The classification was made with the assistance of the developmental agents and local elders. Here, the developmental agent refers to the agricultural extension worker at the smallest administrative unit (locally called kebele) in Ethiopia. Local criteria such as the number of livestock holdings, total size of land holdings, housing standard and annual consumption of food and ~~sold~~ sell crop production were used for this classification. Then, a complete homegarden enumeration was made for the woody species inventory [42]. Finally, a total of 72 homegardens (24 from each altitudinal gradient) were purposively selected for woody species inventory.

Methods of data collection

Woody species with a diameter at breast height (DBH) ≥5cm and height ≥1.5m were identified and their height and DBH were measured. Individuals below this measurement were only counted and registered. For woody branched below 1.3m above the ground, the average DBH was calculated from all branches measured separately [36]. Tree DBH and height were measured using a caliper and a Sunto Clinometer respectively. Tree size beyond the caliper measurement was measured using mater tape and converted to diameter. Tree height difficult to measure with a Sunto Clinometer was measured by a marked range pole. The altitudinal gradient was measured using the Global Positioning System (GPS) Garmin 72. Woody species identification

was made on the field and voucher specimen. A voucher specimen was collected, pressed, dried and identified following [43] and flora of Ethiopia [44-46].

Data analysis

Woody species diversity was analyzed using diversity indices [47-49]. Shannon Wiener diversity index which takes species richness and evenness into consideration was calculated as:

$$H' = -\sum_{i=1}^{s} pi ln pi \ldots\ldots (1)$$

Where: H' = Shannon-Wiener diversity index, s = number of species and Pi = the proportion of individuals of the ith species and ln =s natural logarithm.

A measure of similarity of the abundances of different woody species in the sampled area was analyzed using Evenness/Equitability index.

$$E = \frac{H'}{Hmax} = \frac{H'}{lnS} \text{ with } Hmax = lnS \ldots\ldots (2)$$

Where: E = Evenness (Equitability), Hmax = ln(S) is the maximum level of diversity possible within a given population.

The similarity in species composition of the two sites is computed using Sorensen's Similarity Coefficient (S_s) with values ranging between 0 and 1: 0 indicates complete dissimilarity and 1 complete similarity.

$$Ss = \frac{2a}{2a+b+c} \ldots\ldots (3)$$

Where: Ss = Similarity index, a = number of species common in the two sites; b = number of species recorded only in site one; and c = number of species recorded only in site two.

Importance value index (IVI) is a useful ecological parameter to compare the environmental significance of woody species. IVI was calculated using the sum of relative density, frequency and dominance as:

IVI= Relative density + Relative dominance + Relative frequency ……………………… (4)

Where: $\text{Relative Density (RDe)} = \frac{\text{Stem count of a species}}{\text{Stem count of all species}} * 100$ ………………………….……4.1

$\text{Relative Dominance (RDo)} = \frac{\text{Doninance of a species}}{\text{Dominance of all species}} * 100$ ………………………...….4.2

$\text{Relative Frequency (RF)} = \frac{\text{Frequency of a species}}{\text{Frequency of all species}} * 100$ …………………………….…...4.3

The density of woody species was calculated by converting the total number of individuals encountered in all plots to equivalent number per hectare using the following formula:

$D = N/S$ ………………………………………………………………………………… (5)

Where: D - the density (stems ha^{-1}), N - the number of stems present on the considered surface and S - the area considered in hectare (ha).

Basal area, the area occuied by woody species was calculated for all woody species DBH ≥5cm using the following formula:

Basal area (BA) = $\pi d^2/4$ ……………………………………………………………… (6)

Where: π= 3.14; d = diameter at breast height (m)

Statistical analysis was made using R software version 4.1.3 [50]. Homogeneity and normality of the variance were approved using the LeveneTest and Shapiro-Wilk tests in R statistics. Non-normally distributed data was analyzed using the log transform and the removal of the outlier. The variables were compared using one-way ANOVA (analysis of variance). The relationship between altitudinal gradients and wealth status and the diversity indices was seen using Pearson's correlation coefficient test. The least significant difference (LSD) test was used to compare the level of significance ($P < 0.05$) between each variable.

Results

Floristic composition

~~A~~ In total~~, of~~ 55 woody species belonging to 31 families and 45 genera were recorded in homegarden agroforestry systems. Family Fabaceae is the most dominant having eight species followed by Moraceae, Myrtaceae and Rutaceae each with four species. About 34.5% of the remaining families had only one woody species. About 56.4% of species are indigenous including two endemics (*Erythrina brucei* and *Millettia ferruginea*), and the remaining 43.6% are exotic ~~including two endemics (*Erythrina brucei* and *Millettia ferruginea*)~~ (Appendix 1). The ~~total~~ species richness was 31, 39 and 42 for low, middle and high altitudes, respectively. Wooody species richness increases with increasing wealth status. Accordingly, 27, 40 and 46 species were recorded in poor, medium and rich households, respectively (Table 1).~~Richness increases with wealth status as 27 for poor, 40 for medium and 46 for rich households (Table 1)~~.

Species richness was not significant ($P < 0.05$) between altitudinal gradients whereas it significantly ($P < 0.001$) increased with increasing wealth status (Table 2). The percentage frequency occurrence of ~~the study area~~ woody species lay between 1.4 - 84.7%. *Coffea arabica* (84.7%), *Persea americana* (79.5%), and *Catha edulis* (54.2%) are the most frequently observed ~~recorded woody~~ species. However, about 15 species were observed only once in surveyed homegardens (Appendix 2).

Table 1: Overall woody species richness, genera and family distiribution of woody species across altitudinal gradient and wealth status in homegarden agroforestry systems

**Category**	**Number**	**Richness**	**Genus**	**Family**
**Altitudinal gradient**				
**Low**	24	31	26	21
**Middle**	24	39	36	25
**High**	24	42	37	25
**Total**	72	55	45	31
**Wealth class**				
**Poor**	24	27	24	21
**Medium**	24	40	33	24

**Rich**	24	46	39	27
**Total**	72	55	45	31

Table 2: Species richness, Shannon diversity index and Evenness (Mean ± SE) of woody species across altitudinal gradients and wealth status in southwest Ethiopia

Category	Richness	Shannon	Evenness
**Altitudinal gradients**			
**Low**	7.54^{a}±0.59	1.00^{a}±0.06	0.53^{a}±0.03
**Middle**	7.42^{a}±0.77	1.07^{a}±0.09	0.57^{a}±0.04
**High**	7.75^{a}±0.76	0.97^{a}±0.08	0.55^{a}±0.05
**Mean**	7.57±0.40	1.02±0.05	0.55±0.02
**Wealth status**			
**Poor**	4.75^{c}±0.42	0.84^{b}±0.06	0.61^{a}±0.04
**Medium**	7.58^{b}±0.46	1.07^{a}±0.09	0.53^{a}±0.04
**Rich**	10.38^{a}±0.66	1.14^{a}±0.07	0.50^{a}±0.03
**Mean**	7.57±0.40	1.02±0.05	0.55±0.02

Key- Different letters superscripted along column mean value indicate significant different ($P < 0.05$), SE- standard error

Woody species diversity

The Shannon diversity index and evenness/equitability index were analyzed along an altitudinal gradient and wealth status. Both diversity indicesShannon and evenness were not significant (P < 0.05) between altitudinal gradients. The However, the highest mean Shannon (1.07±0.09) and evenness (0.57±0.04) indexes were observed at middle altitude. The lowest Shannon (0.97±0.08) was recorded at high altitudes whereas the lowest Evenness evenness (0.53±0.03) was recorded at low altitudes. Similar to sSpecies richness and , the mean Shannon diversity index increased with household wealth status, whereas evenness showed the reverse. The Shannon diversity index was significant (P < 0.05) between poor and rich households, while evenness didn't show a significant difference between wealth (Table 2). The Pearson correlation result showed no relation between species richness and altitude, and a positively significant correlation was seen between species richness and wealth status. Furthermore, the Shannon diversity index was significantly and positively correlated to wealth statusThere was a significant positive correlation between the Shannon diversity index and wealth status (Table 3).

Table 3: Pearson correlation between altitudinal gradient, wealth status and woody species diversity in homegarden agroforestry systems

**Category**	**Altitudinal gradients**	**Wealth status**	**Species richness**	**Shannon diversity index**
**Wealth status**	0.000	-	-	-

**Species richness**	0.025	0.674**	-	-
**Shannon diversity index**	-0.028	0.319**	0.470**	-
**Evenness**	0.055	-0.225	-0.270*	0.610**

Note: *- significant different ($P < 0.05$), **- significant different ($P < 0.01$)

Woody species similarity index

The calculated similarity index result indicates that about 51% of the woody species are similar along altitudinal gradients and wealth classes (Fig 2). The highest similarity index was observed between high and middle altitudes (55%) and the lowest (45%) was registered between high and low altitudes. For a wealth class, the highest (60%) similarity was seen between high and medium classes while the lowest (40%) was seen between medium and poor classes (Table 4). On the other hand, 12, seven and three woody species were unique to high, middle and low altitudes respectively, whereas 10, six and two species were recorded for rich, medium and poor households respectively (Fig 2).

Fig 2. Venn diagram representation of the similarity and dissimilarity of woody species across (a) wealth status and (b) altitudinal gradient in homegarden agroforestry systems

Table 4: Woody species similarity index in homegarden agroforestry systems across altitudinal gradient and wealth status

**Altitudinal gradient**	**High**	**Middle**	**Low**

**Middle**	0.55	-	-
**Low**	0.45	0.47	-
**Wealth status**	Rich	Medium	Poor
**Medium**	0.60	-	-
**Poor**	0.44	0.40	-

Woody species density

~~The calculated similarity index result indicates that about 51% of the w~~Woody species stem density was calculated along an altitudinal gradient and wealth status. The mean stem density per homegarden and hectare was 89.06±9.25 and 1236.22±131.42 respectively. The mean stem density was significantly different ($P < 0.001$) between wealth classes. The highest 134.88±18.87 stem density was recorded in the rich followed by medium 87.08±14.35 and poor 45.21±7.38 wealth classes. However, there is no significant difference between stem density and altitudinal gradient at the garden and hectare level (Table 5).

Table 5: Mean (±SE) woody density and basal area distribtution along altitudinal gradients and wealth status in homegarden agroforestry systems

Category	Density/garden	Density/ha	BA/garden	BA/ha
**Altitudinal gradients**				

**Low**	82.04^{a}±14.44	1151.88 ^{a}±140.63	0.64^{a}±0.13	8.97^{a}±1.49
**Middle**	79.88^{a}±12.10	1298.50^{a}±275.63	1.28^{a}±0.31	15.00^{a}±3.49
**High**	105.25^{a}±20.48	1258.29^{a} ±252.41	0.84^{a}±0.28	11.29^{a}±3.79
**Wealth status**				
**Poor**	45.21^{c}±7.38	1654.29^{a}±268.98	0.33^{b}±0.08	10.59 ^{a}±2.71
**Medium**	87.08^{b}±14.35	1046.13^{ab}±169.67	0.90^{ab}±0.27	11.37 ^{a}±1.70
**Rich**	134.88^{a}±18.87	1008.25^{b}±217.68	1.53^{a}±0.29	12.09 ^{a}±3.65

Key: Different letters superscripted along column mean value indicate significant different ($P < 0.05$), SE- standard error

Basal area distribution

The mean basal area was highest at middle altitude followed by high and low altitude respectively, although there was no significant difference between each altitude. Homegarden level basal area was 1.53±0.29 for the rich, 0.90±0.27 for the medium, and 0.33±0.08 for the poor in which a significant difference ($P < 0.01$) was seen between the poor and rich classes (Table 5). However, there was no significant difference in basal area per hectare (m^{2} ha^{-1}) along elevation gradients and wealth status. Finally, the overall basal area distributions of the top ten woody species are presented in Figure 3. *Persea americana* had the highest basal area (886.87 m^{2} ha^{-1}) followed by *Cordia africana* (146.48 m^{2} ha^{-1}), *Coffea arabica* (130.87 m^{2} ha^{-1}), *Mangifera indica* (95.68 m^{2} ha^{-1}), *Catha edulis* (64.98 m^{2} ha^{-1}), *Grevillea robusta* (46.30 m^{2} ha^{-}

^{1}), *Citrus sinensis* (18.01 m^2 ha^{-1}), *Psidium guajava* (16.79 m^2 ha^{-1}), *Carica papaya* (15.69 m^2 ha^{-1}) and *Albizia gummifera* (6.51 m^2 ha^{-1}) (Fig 3).

Fig 3. Basal area distribution (m^2 ha^{-1}) for the top ten woody species in homegarden agroforestry systems at southwest Ethiopia

Importance value index

The overall importance value index in the entire homegarden agroforestry system value lay between 0.20 to 69.06% for less to more important woody species respectively. However, a large number (29) of woody species had an importance value index of less than 1% (Appendix, 1). Figure 4 indicates the importance value index distribution of the top ten woody species. Accordingly, the importance value of these species was presented as *Catha edulis* (69.06%), *Coffea arabica* (51.01%), *Persea americana* (40.84%), *Cordia africana* (18.07%), *Mangifera indica* (15.81%), *Grevillea robusta (11.98%), Psidium guajava (9.31%), Citrus sinensis (9.17%), Ricinus communis (7.97%)* and *Carica papaya* (6.87%) in their descending order (Fig 4).

Fig 4. Importance value index of the top ten woody species across the entire homegarden agroforestry systems

Discussion

Woody species composition and diversity significantly vary between wealth statuses, but not between altitudinal gradients. Species richness and the Shannon diversity index had have shown a significant increasing trend with increasing wealth status. The result was agreed with [14, 51], who reported increased richness and Shannon diversity index with increasing wealth status in

south-central Ethiopia. ~~Similarly, richness and Shannon diversity index increase with increasing wealth status for traditional agroforestry practices in south-central Ethiopia (Tadesse *et al*., 2021; Molla *et al*., 2023).~~ Evenness had shown ~~the~~ a reverse trend with richness in wealth classes. The result indicates that homegardens with few numbers of different species (lower species richness) have a large abundance for each species (higher evenness). However, there was no continuous increasing or decreasing values of diversity indices ~~for~~ seen along altitudinal gradients. Likewise, species richness and diversity ~~of~~ in agroforestry didn't show a parallel relationship with the elevation gradient [52]. *Catha edulis*, *Coffea arabica* and *Persea americana* are the most abundant and frequently recorded woody species. The study conducted by [38] reported that the distributions of farm woody species are determined by the economic or ecological values they provide. Therefore, the main reason for the higher abundance and distribution of these woody species may be due to the farmer's focus on the production of highly economic and ecologically valued woody species in the present study area.

The number of woody species recorded in the present study area is comparable ~~with~~ to the number of woody species (55) reported in the traditional agroforestry practices of Dellomenna district, Ethiopia [53]. The number is lower than 120 trees and shrubs species reported from southern Ethiopia [15] and 60 woody species around Jimma Town, southwestern Ethiopia [39] within similar agroecology. It's also lower than 83 plant species ~~from~~ in eastern Amazonia [54], 138 ~~from~~ in south-central Ethiopia [14], 171 ~~from~~ in central Nepal [55], and 206 ~~from~~ in southern and southwestern Ethiopia [29]. However, it's higher than 53, 36, 49 and 32 woody species reported from Shewarobit district~~, Ethiopia~~ [56], Shashemene district~~, Ethiopia~~ [57], southern Ethiopia [58], and southern Tigray~~, Ethiopia~~ [28] in Ethiopia, respectively. It is also

higher than 32 woody species in western Cameroon [59] and 30 woody species in the mid-highlands of southern Ethiopia [52]~~ from coffee-based agroforestry systems~~.

The present study registered 56.4% indigenous and 43.6% exotic whereas 85% indigenous and 15% exotic were reported in Dellomenna district, southeastern Ethiopia [53]. This inverse result may be observed due to two main reasons. 1) Ecological difference between the two sites as Dellomenna is located in the forest-savannah transition zone of southern Ethiopia and the present study area located in Eastern Afromontane Forests dominated by woody plantation following the expansion of farmland in southwest Ethiopia. 2) Variation in farmers' preference ranking towards the selection of an exotic and native woody species. ~~Comparatively, a high percentage of exotic woody species observed in the study area indicates that the local community gave attention to exotic fruit trees like *Persea americana* and *Mangifera indica* which in the majority of the sampled homegardens. ~~The present finding results agreed with ~~Similarly, ~~the study conducted by [29] ~~also ~~who reported 69.52% indigenous and 31.48% exotic in homegarden agroforestry systems from similar agroecology~~ in Ethiopia~~. Similar to this study, *Erythrina brucei* and *Millettia ferruginea* were also reported as the two endemic woody species in Ethiopia in traditional agroforestry practices [60].

The variation in the woody species similarity index recorded was not only ~~due to~~because of elevation change but also wealth status which was affected by the farmer's homegarden size, extension service, improved seedlings, and labor forces. Likewise, the species similarity index between the sites changed with elevation and woody production [61]. The highest similarity index reported between high and middle altitudes agreed with [52], who reported the highest

species similarity between adjacent elevations in coffee-based agroforestry in Ethiopia. Also, [59] reported the highest woody species similarity due to closed ecological conditions between coffee agroforestry systems and forests in Cameroon.

The mean stem density was significantly increased with wealth status ~~with~~ but there was no significant difference between altitudinal gradients. This indicates that household wealth status determines woody species density in homegarden agroforestry systems. The present study reported the top ten woody species having the highest basal area. Of which, *Persea americana*, an exotic fruit tree species has the highest (61.52%) basal area. However, [51] reported that *Eucalyptus camaldulensis* had the highest (30.03%) basal area among the top ten woody species basal area registered in traditional agroforestry practices of Wondo district, south-central Ethiopia. The distribution indicates that *Cordia africana*, a native endangered tree occupies the second largest basal area which has been recorded on the fourth step among woody species having the highest importance value index. The highest importance value index was recorded for *Catha edulis* ~~followed by *Coffea arabica* and *Persea americana*~~. Similarly, *Catha edulis* was ~~reported~~ recorded as an economically important ~~cash~~ woody crop ~~among woody species of~~ in homegardens ~~in Ethiopia~~ (Gebrehiwot *et al*., 2016). On the contrary, *Eucalyptus camaldulensis* [15, 51], *Faidherbia albida* [34], and *Cordia africana* [40] were reported as the most important woody species in homegarden agroforestry systems in Ethiopia.

Conclusion

Homegarden agroforestry systems ~~of~~ in southwestern Ethiopia are rich in woody species diversity. Households within a rich wealth class had the highest number of woody species

compared to medium and poor wealth classes. Woody species richness was not statistically significant between altitudinal gradients. Shannon diversity index significantly increases with increasing wealth status, with no significant difference between altitudinal gradients. The finding indicates that household economic status determines the diversification of woody species in ecologically related human-managed land use ~~sytems~~systems. Therefore, to improve woody species diversification in homegarden agroforestry, concerned bodies should support the residents through the provision of appropriate supplies~~be provided the necessary materials that support local communities in homegarden agroforestry systems woody species diversification~~. ~~Additionally~~Furthermore, ~~further~~ more investigation will be required to determine the specific variables influencing the ~~could be needed to know details of factors that affect~~ woody species composition and diversity in homegarden agroforestry and its sustainable socio-economic and ecological uses.

References

1. Bishaw, B & Abdelkadir, A. (2003). Agroforestry and Community Forestry for Rehabilitation of Degraded Watersheds on the Ethiopian Highlands. International Conference on African Development Archives. 78. https://scholarworks.wmich.edu/africancentericad-archive/78.
2. Sistla, S.A., Roddy, A.B., Williams, N.E., Kramer, D.B., Stevens, K & Allison, S.D. (2016). Agroforestry Practices Promote Biodiversity and Natural Resource Diversity in Atlantic Nicaragua. *PLoSONE,* **11**(9).

3. Montagnini, F. (2020). The Contribution of Agroforestry to Restoration and Conservation: Biodiversity Islands in Degraded Landscapes. In: J. C. Dagar *et al*. (eds.), Agroforestry for Degraded Landscapes: Recent Advances and Emerging Challenges - Vol. 1, https://doi.org/10.1007/978-981-15-4136-0_15
4. Nair, P.K.R. (1993). An Introduction to Agroforestry. Kluwer Academic Publishers-ICRAF. P. 489.
5. Harvey, C.A & Villalobos, J.A.G. (2007). Agroforestry systems conserve species-rich but modified assemblages of tropical birds and bats. *Biodiversity and Conservation*, **16**: 2257-2292.
6. Bardhan, S., Jose, S., Biswas, S., Kabir, K & Rogers, W. (2012). Homegarden agroforestry systems: an intermediary for biodiversity conservation in Bangladesh. *Agroforest Syst*, **85**:29–34.
7. Jama, B., Elias, E & Mogotsi, K. (2006). Role of agroforestry in improving food security and natural resource management in the drylands: a regional overview. *Journal of the Drylands*, **1**(2): 206-211.
8. Bucheli, V.J.P & Bokelmann, W. (2017). Agroforestry systems for biodiversity and ecosystem services: the case of the Sibundoy Valley in the Colombian province of Putumayo. *International Journal of Biodiversity Science, Ecosystem Services & Management*, **13**(1): 380-397.
9. Nair, P.K.R. (2017). Managed Multi-strata Tree + Crop Systems: An Agroecological Marvel. *Front. Environ. Sci*. **5**:88. doi: 10.3389/fenvs.2017.00088.

10. Rendón-Sandoval, F.J., Casas, A., Moreno-Calles, A.I., Torres-García, I & García-Frapolli, E. (2020). Traditional Agroforestry Systems and Conservation of Native Plant Diversity of Seasonally Dry Tropical Forests. *Sustainability*, **12**: 4600; doi:10.3390/su12114600.

11. Reang, D., Hazarika, A., Sileshi, G.W., Pandey, R., Das, A.K & Nath, A.J. (2021). Assessing tree diversity and carbon storage during land use transitioning from shifting cultivation to indigenous agroforestry systems: Implications for REDD+ initiatives. *Journal of Environmental Management*, **298**: 113470.

12. Endale, Y., Derero, A., Argaw, M & Muthuri, C. (2017). Farmland tree species diversity and spatial distribution pattern in semi-arid East Shewa, Ethiopia. *Forests, Trees and Livelihoods*, **26**(3): 199-214. https://doi.org/10.1080/14728028.2016.1266971

13. van Noordwijk, M., Rahayu, S., Gebrekirstos, A., Kindt, R., Tata, H.L., Muchugi, A *et al*. (2019). Tree diversity as basis of agroforestry. In: van Noordwijk M, ed. Sustainable development through trees on farms: agroforestry in its fifth decade. Bogor, Indonesia: World Agroforestry (ICRAF) Southeast Asia Regional Program. pp 17−44.

14. Tadesse, E., Negash, M & Asfaw, Z. (2021). Impacts of traditional agroforestry practices, altitudinal gradients and households' wealth status on perennial plants species composition, diversity, and structure in south-central Ethiopia. *Agroforestry Systems*, ***95***: 1533-1561.

15. Abebe, T. (2005). Diversity in homegarden agroforestry system of southern Ethiopia. PhD Dissertation, Wageningen Agricultural University. The Netherlands.

16. Beetz, A.E. (2011). Agroforestry: An Overview. The National Sustainable Agriculture Information Service, ATTRA, http://www.attra.ncat.org

17. Leakey, R.R.B. (2017). Definition of agroforestry revisited. In: Multifunctional Agriculture – Achieving Sustainable Development in Africa, RRB Leakey, 5-6, Academic Press, San Diego, California, USA.

18. Korwar, G.R., Prasad, J.V.N.S., Rajeshwara Rao, G., Venkatesh, G., Pratibha, G & Venkateswarlu, B. (2018). Agroforestry as a Strategy for Livelihood Security in the Rainfed Areas: Experience and Expectations. In J.C. Dagar *et al*. (eds.). Agroforestry Systems in India: Livelihood Security & Ecosystem Services. *Advances in Agroforestry*, **10**. https://10.1007/978-81-322-1662-9_5.

19. Dawson, I.K., Guariguata, M.R., Loo, J., Weber, J.C., Lengkeek, A., Bush, D *et al*. (2013). What is the relevance of smallholders' agroforestry systems for conserving tropical tree species and genetic diversity in circa situm, in situ and ex situ settings? A review. *Biodivers Conserv*, **22**(2): 301–324.

20. Schroth G., Fonseca, G.A.B., Harvey, C.A., Gascon, C., Vasconcelos, H.L & Izac, A.M.N. (2004). Agroforestry and Biodiversity Conservation in Tropical Landscapes. Island Press, Washington.

21. Jose, S. (2009). Agroforestry for ecosystem services and environmental benefits: an overview. *Agroforestry Systems*, **76**:1–10.

22. Montagnini, F., Francesconi, W & Rossi, E. (ed.) (2011). Agroforestry as a tool for landscape restoration. Nova Science, New York.

23. Torquebiau, E & Penot, E. (2006). Ecology versus economics in tropical multistrata agroforests. In: B.M. Kumar & P.K.R. Nair (eds). Tropical homegardens: A time-tested example of sustainable agroforestry, 269-282.

24. Mohan, S. (2004). An assessment of the ecological and socioeconomic benefits provided by homegardens: a case study of Kerala, India. University of Florida.

25. Gebremedhin, T., & Mulubrehan, H. (2007). Women in backyards: root crop production and biodiversity management in backyards. A case study in five selected woredas of tigray regional state, Northern Ethiopia. Northern Ethiopia DCG Report No, 50.

26. Maroyi, A. (2009). Traditional homegardens and rural livelihoods in Nhema, Zimbabwe: a sustainable agroforestry system. *International Journal of Sustainable Development & World Ecology*, **16**(1): 1-8.

27. Park, J.H., Woo, S.Y., Kwak, M.J., Lee, J.K., Leti, S & Soni, T. (2019). Assessment of the Diverse Roles of Home Gardens and Their Sustainable Management for Livelihood Improvement in West Java, Indonesia. *Forests*, **10**(970).

28. Eyasu, G., Tolera, M & Negash, M. (2020). Woody species composition, structure, and diversity of homegarden agroforestry systems in southern Tigray, Northern Ethiopia. *Heliyon*, **6**(12), e05500.

29. Kassa, G., Bekele, T., Demissew, S & Abebe, T. (2023). Plant species diversity, plant use, and classification of agroforestry homegardens in southern and southwestern Ethiopia. *Heliyon*, **9**.

30. George, M. V & Christopher, G. (2020). Structure, diversity and utilization of plant species in tribal homegardens of Kerala, India. *Agroforestry systems*, ***94***(1): 297-307.

31. Sood, K.K & Mitchell, C.P. (2009). Identifying important biophysical and social determinants of on-farm tree growing in subsistence-based traditional agroforestry systems. *Agroforest Syst*, **75**:175–187.

32. Bayala, J., Kindt, R., Belem, M & Kalinganire, A. (2011). Factors affecting the dynamics of tree diversity in agroforestry parklands of cereal and cotton farming systems in Burkina Faso. *New Forests*, **41**:281–296.

33. Nyaga, J., Barrios, E., Muthuri, C.W., Öborn, I., Matiru, V & Sinclair, F.L. (2015). Evaluating factors influencing heterogeneity in agroforestry adoption and practices within smallholder farms in Rift Valley, Kenya. *Agriculture, Ecosystems and Environment*, **212**: 106–118.

34. Guyassa, E & Raj, A.J. (2013). Assessment of biodiversity in cropland agroforestry and its role in livelihood development in dryland areas: A case study from Tigray region, Ethiopia. *International Journal of Agricultural Technology*, **9**(4): 829-844.

35. Mekonnen, E.L., Asfaw, Z & Zewudie, S. (2014). Plant species diversity of homegarden agroforestry in Jabithenan District, North-Western Ethiopia. *Int. J. Biodivers. Conserv.* ***6***(4): 301-307.

36. Tadesse, E., Abdulkedir, A., Khamzina, A., Son, Y & Noulèkoun, F. (2019). Contrasting Species Diversity and Values in Home Gardens and Traditional Parkland Agroforestry Systems in Ethiopian Sub-Humid Lowlands. *Forests*, **10**(266).

37. Asfaw, B & Lemenih, M. (2010). Traditional agroforestry systems as a safe haven for woody plant species: a case study from a topo-climatic gradient in South Central Ethiopia. *Forests, Trees and Livelihoods*, ***19***(4): 359-377.

38. Gebrewahid, Y & Abrehe, S. (2019). Biodiversity conservation through indigenous agricultural practices: Woody species composition, density and diversity along an altitudinal gradient of Northern Ethiopia. *Cogent Food & Agriculture,* **5**:1.

39. Wari, B.N., Feyssa, D.H & Kebebew, Z. (2019). Assessment of woody species in agroforestry systems around Jimma Town, Southwestern Ethiopia. *International Journal of Biodiversity and Conservation,* **11**(1): 18-30.

40. Henry, M., Tittonell, P., Manlay, R.J., Bernoux, M., Albrecht, A & Vanlauwe, B. (2009). Biodiversity, carbon stocks and sequestration potential in aboveground biomass in smallholder farming systems of western Kenya. *Agriculture, ecosystems & environment*, ***129***(1-3): 238-252.

41. Legesse, A & Negash, M. (2021). Species diversity, composition, structure and management in agroforestry systems: the case of Kachabira district, Southern Ethiopia. *Heliyon*, **7**(3): e06477.

42. Motuma, T., Zebene, A., Mulugeta, L & Karltun, E. (2008). Woody species diversity in a changing landscape in the south-central highlands of Ethiopia. *Agriculture, Ecosystems and Environment*, **128**: 52–58.

43. Azene B., Birnie, A & Tengnäs, B. (1993). Useful trees and shrubs for Ethiopia: Identification, propagation and management for agricultural and pastoral communities. SIDA's regional Soil Conservation Unit. Ethiopia. P. 474.

44. Edwards, S., Tadesse, M & Hedberg, I. (1995). Flora of Ethiopia and Eritrea, Vol. 2, Part 2, The National Herbarium, Addis Ababa University, Addis Ababa, Ethiopia and Department of Systematic Botany, Uppsala University, Uppsala, Sweden.

45. Hedberg, I & Edwards, S. (1995). Flora of Ethiopia and Eritrea Vol. 7. Poaceae. The National Herbarium, Addis Ababa, Ethiopia, and Department of Systematic Botany, Uppsala, Sweden.

46. Hedberg, I., Kelbessa, E., Edwards, S., Demissew, S & Persson, E. (2006). Flora of Ethiopia and Eritrea Vol. 5. Gentianaceae to Cyclocheilaceae. The National Herbarium, Addis Ababa University, Addis Ababa, Ethiopia and Department of Systematic Botany, Uppsala University, Uppsala, Sweden.

47. Krebs, C.J. (1989). Ecological methodology. New York: Harper Collins Publishers.

48. Kent, M & Coker, P. (1992). Vegetation description and analysis. A practical approach. London: Belhaven Press.

49. Magurran, A.E. (2004). Measuring biological diversity. Malden and Oxford: Blackwell Publishing.

50. R Core Team. (2021). R: A language and environment for statistical computing. R Foundation for Statistical Computing, Vienna, Austria. URL, https://www.R-project.org/.

51. Molla, T., Asfaw, A., Muluneh, M.G & Worku, B.B. (2023). Diversity of woody species in traditional agroforestry practices in Wondo district, south-central Ethiopia. *Heliyon*, **9** e13549. https://doi.org/10.1016/j.heliyon.2023.e13549

52. Tesfay, F., Moges, Y & Asfaw, Z. (2022). Woody species composition, structure, and carbon stock of coffee-based agroforestry system along an elevation gradient in the moist mid-highlands of Southern Ethiopia. *International Journal of Forestry Research, 2022*.

53. Molla, A & Kewessa, G. (2015). Woody Species Diversity in Traditional Agroforestry Practices of Dellomenna District, Southeastern Ethiopia: Implication for Maintaining Native Woody Species. *International Journal of Biodiversity*, **2015**, 13

54. Cardozo, E.G., Muchavisoy, H.M., Silva, H.R., Zelarayán, M.L.C., Leite, M.F.A., Rousseau, G.X *et al*. (2015). Species richness increases income in agroforestry systems of eastern Amazonia. *Agroforestry systems*, **89**: 901-916.

55. Pandey, H.P., Pokhrel, N.P., Luitel, D.R., Acharya, K & Shah, K.K. (2021). Diversity of agroforestry species and uses in two ecological regions: A Case from Central Nepal. *Advances in Agriculture*, ***2021***: 1-9.

56. Woldeamanual, W., Asfaw, Z & Lemessa, D. (2018). The Efect of the Surrounding Landscape and Socioeconomic characteristics on Woody Species Diversity in Homegardens of Shewarobit District, Northeast Ethiopia. *Small-scale Forestry*, https://doi.org/10.1007/s11842-018-9398-1

57. Jegora, T., Asfaw, Z & Anjulo, A. (2019). Woody Species Diversity and Management in Homegarden Agroforestry: The Case of Shashemene District, Ethiopia. *International Journal of Forestry Research*, **2019**, 3697047.

58. Birhane, E., Ahmed, S., Hailemariam, M., Negash, M., Rannestad, M.M & Norgrove, L. (2020). Carbon stock and woody species diversity in homegarden agroforestry along an elevation gradient in southern Ethiopia. *Agroforest Syst*, https://doi.org/10.1007/s10457-019-00475-4.

59. Temgoua, L.F., Etchike, A.B.D., Solefack, M.C.M., Tumenta, P & Nkwelle, J. (2020). Woody species diversity conservation and carbon sequestration potential of coffee agroforestry systems in the Western Region of Cameroon. *Journal of Horticulture and Forestry*, **12**(2): 35-48.

60. Bajigo, A & Tadesse, M. (2015). Woody species diversity of traditional agroforestry practices in Gununo watershed in Wolayitta zone, Ethiopia. *Forest Research*, **4**(4): 2168-9776.

61. Chemeda, B.A., Wakjira, F.S & Hizikias, E.B. (2022). Tree diversity and biomass carbon stock analysis along altitudinal gradients in coffee based agroforestry system of Western Ethiopia. *Cogent Food & Agriculture*, **8**(1): 2123767.

62. Gebrehiwot, M., Elbakidze, M., Lidestav, G., Sandewall, M., Angelstam, P & Kassa, H. (2016). From self-subsistence farm production to khat: driving forces of change in Ethiopian agroforestry homegardens. *Environmental Conservation*, **43**(3): 263-272.

Appendix 1: List of woody species scientific, local and family name, and their origin in homegarden agroforestry systems of southwest Ethiopia

Nº	Scientific name	Local name	Family name	Origin
1	*Acacia abyssinica* Hochst. ex Benth.	Sondii	Fabaceae	I
2	*Albizia gummifera* (J. F. Gmel.) C.A. Sm.	Ambabbessa	Fabaceae	I
3	*Albizia schimperiana* Oliv.	Ambabbessa arba	Fabaceae	I
4	*Allophylus abyssinicus* (Hochst.) Radikofer	Sehoo	Sapindaceae	I
5	*Annona senegalensis* pers.	Gishxaa	Annonaceae	I
6	*Artocarpus heterophyllus*	Jakifiruttii	Moraceae	E

7	*Azadirachta indica* A.Juss.	Niimii	Meliaceae	E
8	*Borassus aethiopum* Mart.	Meexxii	Arecaceae	I
9	*Brucea antidysenterica* J.F.Miller	Qomonyoo	Simaroubaceae	I
10	*Calpurnia aurea* (Ait.) Benth.	Ceekaa	Fabaceae	E
11	*Carica papaya* L.	Papaayee	Caricaceae	I
12	*Casimiroa edulis* LaLava	Kashimiirii	Rutaceae	E
13	*Casuarina equisetifolia*	Shuwashuwee	Casuarinaceae	E
14	*Catha edulis* (Vahl) Forssk. ex Endl.	Caatii	Celastraceae	I
15	*Citrus aurantifolia* (Christm.) Swingle	Loomii	Rutaceae	E
16	*Citrus medica*	Maraaraa	Rutaceae	E
17	*Citrus sinensis*	Burtukaana	Rutaceae	E
28	*Coffea arabica* L	Buna	Rubiaceae	I
19	*Cordia africana* Lam.	Waddeessa	Boraginaceae	I
20	*Croton macrostachyus* Hochst ex Del.	Makkannisa	Euphorbiaceae	I
21	*Cupressus lusitanica*	Gattira faranjii	Cupressaceae	E
22	*Dovyalis abyssinica* (A. Rich.) Warb.	Koshimii	Flacourtiaceae	I

23	*Dracaena fragrans*	Omoo	Dracaenaceae	I
24	*Dracaena steudneri* Engl.	Bubbiftuu	Dracaenaceae	I
25	*Duranta erecta* L.	Ababaa	Verbenaceae	E
26	*Ehretia cymosa* Thonn.	Ulaagaa	Boraginaceae	I
27	*Erythrina abyssinica* Lam. ex DC.	Waleensuu	Fabaceae	I
28	*Erythrina brucei* Schweinf.	Beroo	Fabaceae	I®
29	*Eucalyptus camaldulensis*	Bargamo diimaa	Myrtaceae	E
30	*Eucalyptus citriodora* Hook.	Bargamo	Myrtaceae	E
31	*Eucalyptus globules* Labill.	Bargamo adii	Myrtaceae	E
32	*Ficus thonningii* Blume	Dambii	Moraceae	I
33	*Ficus sur* Forssk.	Harbuu	Moraceae	I
34	*Grevillea robusta* A.Cunn. ex R.Br.	Giraavillaa	Proteaceae	E
35	*Jacaranda mimosifolia* D.Don	Jakarandaa	Bignoniaceae	E
36	*Maesa lanceolata* Forssk.	Abbayyii	Myrsinaceae	I
37	*Malus domestica*	Appilii	Rosaceae	E
38	*Mangifera indica* L.	Maangoo	Anacardiaceae	E

39	*Millettia ferruginea* (Hochst.) Baker	Askiraa	Fabaceae	I®
40	*Moringa oleifera*	Mooriingaa	Moringaceae	E
41	*Morus alba* L.	Goraa	Moraceae	E
42	*Ocimum lamiifolium* Hochst. ex Benth	Damakasee	Lamiaceae	I
43	*Olea europaea* (Wall. ex DC.) Cifferri	Ejersa	Oleaceae	I
44	*Olea welwitschii* (Knobl.) Gilg & Schellenb.	Bayaa	Oleaceae	I
45	*Persea americana* Mill.	Avokaadoo	Lauraceae	E
46	*Pouteria campechiana*	Eggifiruttii	Sapotaceae	E
47	*Prunus persica* (L.) Batsch	Kuukkoo	Rosaceae	E
48	*Psidium guajava* L.	Zetunaa	Myrtaceae	E
49	*Rhamnus prinoides* L'Herit	Geeshoo	Rhamnaceae	I
50	*Ricinus communis* L.	Qobboo	Euphorbiaceae	I
51	*Sapium ellipticum* (Hochst) Pax.	Bosoqa	Euphorbiaceae	I
52	*Sesbania sesban*	Sasbaniyaa	Fabaceae	I
53	*Spathodea campanulata*	Ispaatoodaa	Bignoniaceae	E
54	*Vernonia amygdalina* Del.	Eebicha	Asteraceae	I

55	*Vernonia auriculifera* Heirn.	Reejjii	Asteraceae	I

Key: Origin: I- Indigenous, E- Exotic, ®- Endemic species

Appendix 2: Percentage frequency (%Freq), relative dominance (Rdo), relative density (Rde), relative frequency (RF) and importance value index (IVI) of woody species across the entire homegarden agroforestry systetms

N/S	Scientific Name	% Freq	Rdo	Rde	RF	IVI
1	*Catha edulis*	54.2	7.4	54.5	7.1	69.1
2	*Coffea arabica*	84.7	10.6	29.3	11.1	51.0
3	*Persea americana*	79.2	27.5	3.0	10.4	40.8
4	*Cordia africana*	44.4	11.2	1.1	5.8	18.1
5	*Mangifera indica*	44.4	9.0	1.0	5.8	15.8
6	*Grevillea robusta*	36.1	6.3	1.0	4.7	12.0
7	*Psidium guajava*	37.5	3.8	0.6	4.9	9.3
8	*Citrus sinensis*	34.7	3.9	0.7	4.6	9.2
9	*Ricinus communis*	40.3	1.2	1.5	5.3	8.0
10	*Carica papaya*	20.8	3.7	0.5	2.7	6.9

11	*Albizia gummifera*	27.8	2.4	0.4	3.6	6.4
12	*Vernonia auriculifera*	30.6	0.0	1.0	4.0	5.0
13	*Cupressus lusitanica*	18.1	1.2	0.7	2.4	4.3
14	*Annona senegalensis*	18.1	1.3	0.5	2.4	4.1
15	*Duranta erecta*	23.6	0.0	0.7	3.1	3.8
16	*Croton macrostachyus*	13.9	0.8	0.3	1.8	2.9
17	*Erythrina brucei*	11.1	0.8	0.3	1.5	2.6
28	*Vernonia amygdalina*	12.5	0.7	0.1	1.6	2.4
19	*Jacaranda mimosifolia*	6.9	1.3	0.1	0.9	2.3
20	*Casimiroa edulis*	11.1	0.7	0.2	1.5	2.4
21	*Sesbania sesban*	12.5	0.1	0.4	1.6	2.2
22	*Albizia schimperiana*	6.9	0.8	0.1	0.9	1.8
23	*Millettia ferruginea*	4.2	1.1	0.1	0.6	1.7
24	*Calpurnia aurea*	9.7	0.1	0.4	1.3	1.7
25	*Rhamnus prinoides*	6.9	0.0	0.2	0.9	1.1
26	*Ehretia cymosa*	4.2	0.4	0.1	0.6	1.0

27	*Acacia abyssinica*	1.4	0.7	0.1	0.2	0.9
28	*Malus domestica*	5.6	0.0	0.3	0.7	1.0
29	*Erythrina abyssinica*	5.6	0.1	0.1	0.7	0.9
30	*Citrus aurantifolia*	5.6	0.1	0.1	0.7	0.9
31	*Olea europaea*	4.2	0.2	0.1	0.6	0.8
32	*Casuarina equisetifolia*	2.8	0.4	0.0	0.4	0.8
33	*Eucalyptus citriodora*	2.8	0.2	0.1	0.4	0.7
34	*Eucalyptus globulus*	2.8	0.2	0.1	0.4	0.6
35	*Dovyalis abyssinica*	2.8	0.1	0.1	0.4	0.5
36	*Azadirachta indica*	2.8	0.1	0.0	0.4	0.5
37	*Olea welwitschii*	2.8	0.1	0.0	0.4	0.5
38	*Citrus medica*	2.8	0.1	0.0	0.4	0.5
39	*Dracaena steudneri*	1.4	0.3	0.0	0.2	0.5
40	*Artocarpus heterophyllus*	6.9	0.2	0.0	0.2	0.5
41	*Maesa lanceolata*	2.8	0.1	0.0	0.4	0.5
42	*Moringa oleifera*	1.4	0.2	0.0	0.2	0.4

43	*Morus alba*	2.8	0.0	0.0	0.4	0.4
44	*Borassus aethiopum*	1.4	0.2	0.0	0.2	0.4
45	*Allophylus abyssinicus*	1.4	0.2	0.0	0.2	0.4
46	*Pouteria campechiana*	1.4	0.1	0.0	0.2	0.3
47	*Ficus sur*	1.4	0.1	0.0	0.2	0.3
48	*Dracaena fragrans*	1.4	0.1	0.1	0.2	0.3
49	*Ficus thonningii*	1.4	0.1	0.0	0.2	0.3
50	*Eucalyptus camaldulensis*	1.4	0.1	0.0	0.2	0.3
51	*Prunus persica*	1.4	0.1	0.0	0.2	0.3
52	*Spathodea campanulata*	1.4	0.1	0.0	0.2	0.3
53	*Sapium ellipticum*	1.4	0.1	0.0	0.2	0.3
54	*Ocimum lamiifolium*	1.4	0.0	0.0	0.2	0.2
55	*Brucea antidysenterica*	1.4	0.0	0.0	0.2	0.2

Response to the major questions raised by the editors or reviewers

1. Map of the study area has been created using shapefiles acquired for free at the Ethiopian Mapping Agency. The Digital Elevation Model (SRTM, 30m resolution) can be accessed for free at EarthExplorer (usgs.gov)

2. The title of the manuscript was writted as commented using a track change

3. Unnecessary italic format in abstract and tables was removed, and there are no italic words and sentences except for writing scientific names

4. All authorities' names were corrected for woody species' scientific names (appendix 1)

5. All manuscript body format was corrected following PLOS ONE guidelines

6. The introduction, results, discussions and conclusion were re-visited and improved

7. Language in the manuscript was revised to meet the needs of international publishing standards

8. Unnecessary numbers in the brackets were removed

9. Manuscript line numbers were assigned to adjust the location of the text for further comments
