# Supplementary material for: Woody species composition and diversity of agroforestry homegardens along altitudinal gradient in southwest Ethiopia
Source: PLoS One. 2025 Jan 7;20(1):e0313520. doi: 10.1371/journal.pone.0313520 (PMC11706438; doi:10.1371/journal.pone.0313520)
Supplement: Attachment — Submitted filename: Response to Reviewers.doc [file pone.0313520.s003.doc]

**Response to the major questions raised by the editors or reviewers**

1. Map of the study area has been created using shapefiles acquired for free at the Ethiopian Mapping Agency. The Digital Elevation Model (SRTM, 30m resolution) can be accessed for free at [EarthExplorer (usgs.gov)](https://earthexplorer.usgs.gov/)
2. The title of the manuscript was writted as commented using a track change
3. Unnecessary italic format in abstract and tables was removed, and there are no italic words and sentences except for writing scientific names
4. All authorities’ names were corrected for woody species’ scientific names (appendix 1)
5. All manuscript body format was corrected following PLOS ONE guidelines
6. The introduction, results, discussions and conclusion were re-visited and improved
7. Language in the manuscript was revised to meet the needs of international publishing standards
8. Unnecessary numbers in the brackets were removed
9. Manuscript line numbers were assigned to adjust the location of the text for further comments
