# Supplementary material for: Woody species composition and diversity of agroforestry homegardens along altitudinal gradient in southwest Ethiopia
Source: PLoS One. 2025 Jan 7;20(1):e0313520. doi: 10.1371/journal.pone.0313520 (PMC11706438; doi:10.1371/journal.pone.0313520)
Supplement: Attachment — Submitted filename: Response to Reviewers (2nd round).doc [file pone.0313520.s005.doc]

**Response to the questions raised by the reviewers**

We have seen all the comments given by the reviewers. It is a clear and constructive idea to improve the quality of the manuscript. Therefore, we address all the comments raised by the reviewers as ordered by the journal editor-in-chief. Finally, we inform you that we have no problems with the given comments and have prepared the revised version and attached it in a separate file (as Manuscript).
